# Potential predictive and therapeutic applications of small extracellular vesicles-derived circPARD3B in osteoarthritis

**DOI:** 10.3389/fphar.2022.968776

**Published:** 2022-10-19

**Authors:** Zhiguo Lin, Yeye Ma, Xiaoying Zhu, Siming Dai, Wentian Sun, Wenjing Li, Sijia Niu, Maolin Chu, Juan Zhang

**Affiliations:** ^1^ Department of Rheumatology, The First Affiliated Hospital, Harbin Medical University, Harbin, China; ^2^ Department of Urology, The Second Affiliated Hospital, Harbin Medical University, Harbin, China

**Keywords:** circPARD3B, SIRT1, VEGF, osteoarthritis, small extracellular vesicles (sEV)

## Abstract

**Background:** Heterogeneous phenotypes that display distinct common characteristics of osteoarthritis (OA) are not well defined and will be helpful in identifying more customized therapeutic options for OA. Circular RNAs (circRNAs) have attracted more and more attention due to their role in the progression of OA. Investigating the role of circRNAs in the pathogenesis of OA will contribute to the phenotyping of OA and to individualized treatment.

**Methods:** Small extracellular vesicles (sEV) were isolated from serum samples from patients with OA of different stages and sEV-derived circPARD3B was determined using RT-qPCR analysis. CircPARD3B expression in a stimulated coculture that included OA fibroblast-like synoviocytes (OA-FLS) as well as human dermal microvascular endothelial cells (HDMECs), plus the effects of circPARD3B on the expression of vascular endothelial growth factor (VEGF) long with angiogenic activity, were evaluated *in vitro*. Based on bioinformatics analysis and luciferase reporter assay (LRA), MiR-326 and sirtuin 1 (SIRT1) were found to be interactive partners of circPARD3B. Mesenchymal stem cells (SMSCs) overexpressing circPARD3B were constructed and SMSCs-derived sEV with overexpressed circPARD3B (OE-circPARD3B-SMSCs-sEV) were obtained to explore the effect of the intervention of circPARD3B combined with SMSCs-sEV-based therapy *in vitro* and in a OA model induced by collagenase *in vivo*.

**Results:** Serum sEV-linked circPARD3B was indentified to be significantly decreased in the inflammatory phenotype of OA. Overexpression of circPARD3B was found to inhibit the expression of VEGF, as well as the angiogenesis induced by VEGF in a IL-1β stimulated the co-culture of OA-FLS as well as HDMECs. CircPARD3B is directly bound to miR-326. SIRT1 was considered a novel miR-326 target gene. OE-circPARD3B-SMSCs-sEV significantly reduced VEGF expression in coculture of OA-FLS and HDMECs. Injection of OE-circPARD3B-SMSCs-sEV could also reduce synovial VEGF; additionally, it could further ameliorate OA in the mouse model of OA *in vivo*.

**Conclusion:** Serum sEV circPARD3B is a potential biomarker that enables the identification of the inflammatory phenotype of patients with OA. Correspondingly, intracellular transfer of circPARD3B through OE-circPARD3B-SMSCs-sEV could postpone disease progression through a functional module regulated angiogenesis of circPARD3B-miR-326-SIRT1, providing a novel therapeutic strategy for OA.

## Introduction

Osteoarthritis (OA) is the most common joint disease, but its cause remains unknown (Walsh, 2004). Currently, therapeutic agents have focused mainly on symptomatic relief of pain. OA frequently progresses to the stage of total joint replacement surgery (Dixon et al., 2004), partly attributed to heterogeneity. OA may occur due to posttraumatic, genetic, metabolic, biomechanical and inflammatory factors, and it has been proposed that OA is a syndrome comprised of multiple distinct phenotypes rather than a single disease (Arden et al., 2015). Identifying OA phenotypes would allow targeted treatments for specific subgroups, ultimately making the treatments more effective. However, at present, this ideal solution is still in the stage of exploration, with the situation that the OA phenotypes are usually limited by imaging or serum protein molecular expression, and treatment aimed at different phenotypes defined by biomarrkers has rarely been reported.

OA is linked with fibrosis, articular cartilage loss, osteophyte formation, and subchondral bone remodeling. Traditionally, arthritis is classified as either an inflammatory disease (such as rheumatoid arthritis [RA]) or a non-inflammatorydisease (OA). However, synovial inflammation has been increasingly recognized as a critical characteristic of OA. And the angiogenesis accompanying synovitis (Haywood et al., 2003), which may be largely driven by angiogenic factors such as vascular endothelial growth factor (VEGF) secreted from endothelial cells and fibroblasts. In addition, as a feature of RA, pannus is identified in OA as well (Bonnet and Walsh, 2005). Fibroblast-like type B synoviocytes oftern congregate at the junction of cartilage and pannus, producing matrix metalloproteinases (MMPs [especially 13]) to degrade the articular cartilage (Yuan et al., 2004). OA is not typically characterized by bony erosion, despite the adverse effects of pannus on cartilage homeostasis, as well as its contribution to cartilage damage. The growth of the pannus along with its cartilage-degrading activity could be strengthened by inflammation and mediated by IL-1β. Therefore, the role of inflammation-induced angiogenesis in OA synovium needs to be recognized and may pave the way for more effective therapies.

Circular RNAs (circRNAs), a novel class of endogenous covalently linked RNA molecules, were characterized by a closed continuous loop. CircRNAs are abundant, widespread, and tissue specific (Kristensen et al., 2019). CircRNAs have primary played a role as microRNA (miRNA) sponges. As evolutionarily conserved small noncoding RNAs (ncRNAs), miRNAs could inhibit gene expression by binding the base with the 3′untranslated region (3′-UTR) of mRNAs (Kristensen et al., 2019). As reported, circRNAs are related with various pathological progrssions and possibly new therapeutic targets in human diseases. However, circRNAs’ effect on the pathogenesis of diseases such as OA, especially in the development of disease of different phenotypes, remains unclear. Additionally, circRNAs are enriched and stable in sEV, which are nano-sized membrane-bound vesicles and provide a novel method of transferring effector messages between cells, indicating that circRNAs could be promising biomarkers for complicated diseases (Fanale et al., 2018). The method for executing the application of circRNA as a combination of biomarker and therapeutic targets remains undefined and will be of great significance.

In this study, hsa_circ_0008172 (circPARD3B) was obviously down-regulated in the synovial tissues of OA. Overexpression of circPARD3B decreased the level of vascular endothelial growth factor (VEGF) from the coculture of OA-FLS, as well as vascular endothelial cells under inflammatory condition. More interestingly, serum small extracellular vesicles (sEV) -derived circPARD3B was found to be significantly increased in the inflammatory phenotype of OA. Therefore, sEV-linked circPARD3B could be a novel biomarker to define one OA phenotype, and more importantly, bioengineered circPARD3B modified sEV from mesenchymal stem cells (MSCs) provided an effective method of individualization combined with precision treatment of OA. It might possess a great potential for the treatment of diseases including OA and so on.

## Materials and methods

### Cell isolation and culture

Arthroscopic surgeries for arthrotrauma were performed and synovial tissue from healthy donors (mean ages 42 years, range 32–50 years) were obtained. Synovial MSCs (SMSCs) and fibroblast-like synovial cells of normal humans (NH-FLS) were isolated, expanded and identified as previously reported (Zhang et al., 2021b). Synovium was obtained from the knees of patients with OA in total knee replacement surgery in the Department of Orthopaedics, the First Affiliated Hospital of Harbin Medical University (HMU). Furthermore, the diagnosis of knee OA complied with the criteria of the American College of Rheumatology (Altman et al., 1986). Fibroblast-like synovial cells from patients with OA (OA-FLS) were obtained as reported (Alvaro-Gracia et al., 1990). Human dermal microvascular endothelial cells (HDMECs) were purchased from ScienCell Research Laboratories (Carlsbad, CA, United States) and cultured in endothelial cell growth medium (EGM) −2 (Procell Life Science&Technology, Wuhan, China). Finally, cells at passages 4-7 were adopted in this study.

### OA-FLS and HDMECs co-culture as well as *in vitro* angiogenic activity *testing*

The coculture system of OA-FLS and HDMECs co-culture system was established in accordance with a modified protocol (Zhang et al., 2017). In a transwell system, OA-FLS (4 × 10^5^ cells/ml) were seeded in the lower chambers but HDMECs (ranging from 3 × 10^5^ cells/ml) were seeded in the upper chambers, followed by incubation with or without IL-1β (10 ng/ml, Peprotech, United States) to mimic the inflammatory microenvironment for 48 h. The fresh supernatants were then obtained for subsequent tube formation assays (TFA), *ex vivo* aortic ring assay (ARA) of angiogenesis as well as the detection of VEGF concentration as previously described (Zhang et al., 2021a). The mRNA and protein expression were measured in OA-FLS and HDMECs co-culture using quantitative real-time PCR (RT-qPCR) along with western blot analysis (WB) (Chu and Zhang, 2018).

### Bioinformatics analysis

The bioinformatics databases TargetScan (http://www.targetscan.org/) and CircInteractome (https://circinteractome.nia.nih.gov/) were applied to predict the miRNA target of circPARD3B. TargetScan (http://www. Targetscan. org/) and Starbase (http://starbase.sysu.edu.cn/) were used to predict the miR-326 mRNA target.

### Virus infection as well as cell transfection

The preparation of Adenovirus vectors encoding circPARD3B (circPARD3B-OE), SIRT1 (SIRT1-OE) along with GFP control (Hanbio Biotechnology, Shanghai, China) and the establishement of SMSCs/OA-FLS overexpression was conducted as previously described (Zhang et al., 2021b). To overexpress circPARD3B, circRNA PARD3B vector was synthesized. We inserted the PARD3B exons 11–15 along with endogenous flanking sequence (1 kb upstream) into pcDNA3.1, and then we copied part of the upstream flanking sequence and inserted it in an inverted orientation downstream. CircPARD3B-ir without the downstream reverse sequence was used as a negative control. All vetors were finally cloned into the Adenovirus Expression System. All constructs were verified by sequencing. A predesigned siRNA targeting human SIRT1 (SIRT1-KD) was purchased from GenePharma (Shanghai, China), followed by transfection into OA-FLS by Lipofectamine 2000 (Invitrogen, Carlsbad, CA, United States). The vectors that regulate/decrease relative miRNA expression (including miRNA mimics and miRNA inhibitors) were designed and constructed (RiboBio, Guangzhou, China).

### RNA fluorescence in situ hybridization (FISH)

FISH detection using FISH Kits (RiboBio, Guangzhou, China) was conducted as reported (Zhang et al., 2021a). OA-FLS were incubated in hybridization buffer using a circRNA probe labelled by CY3 (RiboBio, Guangzhou, China) (Supplementary Table S1). Additionally, the localization of circRNA insided the cells was confirmed by a TCS SP8 X laser confocal microscope (LEICA).

### Pull-down assays with biotinylated DNA probes

Biotinylated DNA probes complementary to the backsplice sequence of circPARD3B was designed and synthesized by GenePharma (Shanghai, China). In order to generate probe-coated magnetic beads, the 3’ end biotin-coupled DNA probes were incubated with streptavidin-conjugated magnetic beads (Invitrogen, United States). OA-FLS lysates were incubated with probe-coated beads and RNeasy Mini Kit (QIAGEN) was used to extract RNA complexes bound to the beads. 10% of the lysates were aliquoted for input assay. Level of circPARD3B was determined by qPCR. Following the circPARD3B probe pull-down assay, miRNAs were extracted, and the levels of candidate miRNAs were measured by qPCR. The oligonucleotide sequences were shown in Supplementary Table S1.

### Pull-down assays with biotinylated miRNA

Using Lipofectamine RNAiMax (Life Technologies), OA-FLS were transfected with biotinylated miR-326 mimics or mutant (RiboBio, Guangzhou, China, 50 nM). After 48 hours, cells were incubated with a lysis buffer (Ambion). The lysates were further incubated with streptavidin-conjugated magnetic beads (Invitrogen, United States). 10% of the cell lysates were aliquot for input. RNAs bound to the beads were extracted using the RNeasy Mini Kit (QIAGEN) for the subsequent analysis.

### Luciferase reporter assay (LRA)

LRA was carried out based on modified methods (Zhang et al., 2021b). In summary, OA-FLS were transfected with miR-326 mimic (RiboBio, China) or control mimic combined with a luciferase (luc) reporter or empty vector; In addition, cells were transfected with pcDNA3.1-circPARD3B along with its mutant (Addgene) as well. Subsequently, the SIRT1 gene 3′-UTR containing either miR-326 target sites or their mutant sequences were then inserted into the pGL3 promoter vector, followed by the tranfection of MiR-326 mimics/inhibitors, as well as corresponding negative controls, into OA-FLS using a riboFECT TM CP Kit (RiboBio, China) that complies with the protocol from the manufacturer. The activity of luc was detected by a dual-gel luc assay system (Promega, Madison, WI) with a Cytation 5 cell imaging multi-mode reader (BioTek) 24 h after transfection.

### Study participants for the evaluation of serum sEV circPARD3B

Forty-five patients who had been diagnosed with primary knee OA according to the criteria of the American College of Rheumatology (Altman et al., 1986) were selected from the HMU First Affiliated Hospital between July 2019 and September 2021. Patients with 1 knee joint with soft tissue swelling were eligible. Clinical inflammation, ultrasound abnormalities, and radiographic erosions did not have to be present in the same joint. Exclusion criteria were patients with another autoimmune or inflammatory rheumatic disease, infectious OA, posttraumatic OA, crystal arthritis, knee avascular necrosis, history of knee arthroplasty or intraarticular injection within the previous 4 weeks, treatment of steroids or immunomodulating drugs within the previous 90 days. Synovitis ultrasound signals along with its accompanied symtoms (such as synovial hypertrophy/fluid), changes in joints, and damages to the eniscus were semi-quantitatively evaluated (0–3) according to reported standardized US scoring systems for OA (Bruyn et al., 2016). Briefly, the suprapatellar, parapatellar (medial and lateral) recesses, along with the facets (medial and lateral) of the trochlear cartilage area were scanned for every knee. To detect and score the lesions, the following planes were scanned, including the longitudinal plane for the suprapatellar recess, the femorotibial (medial and lateral) space, the medial horn of the medial meniscus, the transverse plane to the patella for the parapatellar recesses, as well as the transverse plane for the trochlear articular cartilage (on maximally flexed knee joints). Thirty healthy subjects with the same age, sex, and ethnicity were used as non-OA controls. To assess the severity of knee OA, posteroanterior radiographs of the knees were taken at 20° flexion. Knee radiographs were scored using the Kellgren-Lawrence (K&L) scale (Scott et al., 1993). The demographic and clinical characteristics of the study subjects are shown in Supplementary Table S2.

### Isolation and confirmation of sEV

Based on differential centrifugations, sEV were isolated from the SMSCs culture medium complying with our published protocol (Zhang et al., 2021b). We obtained serum from each of the two groups (healthy controls and patients with OA) and serum sEV were isolated from 1 ml serum aliquots from each subject using a modified differential ultracentrifugation protocol (Gardiner et al., 2016; Langevin et al., 2019). The information of the patients enrolled in this study was collected and shown in Figure 5C, Supplementary Table S2.

The confirmation of sEV was performed using transmission electron microscopy (TEM) (Libra 200 FE, Zeiss, Germany). DLS detection was carried out using a nanoparticle size analyzer (Zetasizer Nano ZS90) and WB analysis.

### Incubation and labeling of SMSCs-sEV based on coculture of OA-FLS and HDMECs

OA-FLS (4 × 10^5^ cells per ml) and HDMECs (3 × 10^5^ cells per ml) were co-cultured in the aforementioned transwell apparatus, followed by incubation in cell growth medium with depleted sEV (SBI). Subsequently, SMSCs-derived sEV (SMSCs-sEV, 100 µg) were put into the coculture. After incubation in fetal bovine serum (FBS), 1% Dulbecco modified Eagle medium for 48 h, the supernatants were obtained and centrifuged (120,000 × g, 120 min) to remove sEV. Subsequently, TFA together with *ex vivo* ARA of angiogenesis was performed under the management of culture supernatants. The remaining supernatants were further analyzed for VEGF using a commercial ELISA kit (R&D Systems). Furthermore, the expression of mRNA and protein in the OA-FLS and HDMECs coculture was measured by RT-qPCR as well as WB, respectively.

The PKH67 Green Fluorescent Cell Linker Kit (Sigma) was applied for the staining of the sEV as previously described (Zhang et al., 2021b). Briefly, sEV were mixed and labeled with 100 μl PKH67 dye solution for 5 min, followed by incubation with recipient OA-FLS or HDMECs for 6 h using a laser confocal microscope (LEICA TCS SP8) before imaging.

### RNA isolation as well as RT-qPCR

After extracted from cultured cells along with sEV using TRIzol reagent (Invitrogen) based on the published method (Zhang et al., 2021b), the total RNA was further incubated with ribonuclease R (RNase R) (Geneseed Biotech, Guangzhou, China) before detection of circRNA. To detect mRNA and circRNA, cDNA was synthesized with PrimeScript RT Master Mix (Takara Biotechnology, Dalian, China). Subsequently, qRT–PCR was performed using iTaq Universl SYBR Green Supermix (Bio–Rad). To determine miRNA, cDNA was synthesized using a riboSCRIPTTM reverse transcription kit (RiboBio, Guangzhou, China). Subsequently, qRT–PCR was performed using an Applied Biosystems TaqMan MicroRNA Assay Kit. Primers are listed in Supplementary Table S1. GAPDH and U6 were used as internal controls. The relative expression of circRNA, miRNA, and mRNA was compared with the internal controls and analyzed by the 2^−ΔΔCt^ method.

### WB analysis

Protein extraction and WB analysis were carried out as previously reported (Chu and Zhang, 2018). Furthermore, antibodies against specific proteins, including SIRT1 (Abbkine, Wuhan, China, ABP0119), VEGF (Abcam, ab52917), MMP13 (Abbkine, Wuhan, China, ABP51805), CD63 (Abcam, ab134045) as well as TSG101 (Abcam, ab125011), were adopted.

### Collagenase-induced OA mice model

Male C57BL/6 mice (12 weeks old, SPF, Second Affiliated Hospital of HMU Animal Centre, Harbin, China) were obtained to establish the collagenase-induced OA model with some modifications (Van Der Kraan et al., 1990). Treatment of collagenase type VII could result in knee joint instability by damaging the cruciate plus collateral ligaments. Finally, chronic synovial activation as well as cartilage destruction were induced, representing an OA-like phenotype. Briefly, 5 U collagenase type VII (Sigma-Aldrich) was injected intraarticularly into the right knee on days 0 and 2. The mice were then grouped as follows (n = 6 for each group): OA control, SMSCs-sEV, Vector-SMSCs-sEV, OE-circPARD3B-SMSCs-sEV (mice with OA treated with sEV [100 µg] in PBS [10 µl], twice a week) and a normal control group (NC) (without collagenase injection). Intraarticular injection in mice with OA were administered from day 7 to day 42. The NC group and the OA control group were administered with equal volume of PBS through injection.

### Micro-computed tomography (micro-CT) imaging

On day 42, mice were scanned for the 3D structure based on micro-CT imaging (Quantum GX, Perkin Elmer, Waltham, United States) as previously described (Zhang et al., 2021a). Unbalanced bone reconstruction was usually characterized by an increase in osteophyte production.

### Histological examination and immunohistochemistry (IHC)

The knee joints of the mice were embedded in paraffin and then cut into slices (5-μm in thickness) for subsequent histological examination. Furthermore, safranin O/fast green staining was performed to assess proteoglycan loss, and an OARSI scoring system was used to grade the severity of cartilage degeneration as previously described (Yang et al., 2021). To estimate synovial inflammation and bone erosion, hematoxylin and eosin staining (H&E) staining was performed and a scoring system was used to quantitatively assess the severity of arthritis in accordance with the published method (Zhang et al., 2017).

IHC analysis was carried out as previously described (Zhang et al., 2021a). After incubation with specific antibodies against SIRT1, VEGF, or MMP13 (Wanleibio, Shenyang, China), knee joint section slides were further incubated using a polymer-HRP detection system (PV9001, ZSGB-BIO) and visualized using a diaminobenzidine peroxidase substrate kit (ZLI-9017, ZSGB-BIO). The evaluation of synovial areas were conducted under a microscope (LEICA DMi8, Germany, 100×). The average integrated optical density (IOD) of 3 areas randomly selected from the acquired images was analyzed by Image-Pro Plus 6 (Media Cybernetics, Inc.).

### Statistical analysis

Statistical analysis was performed using SPSS 22.0. Data were described as means ± Standard Error of Means (SEM). Descriptive statistics are shown as mean ± standard deviation. Statistical analysis was performed with Student’s t test, one-way analysis of variance (ANOVA), or chi-square test, as appropriate. Multiple group comparisons were performed by one-way or two-way analysis of variance followed by Bonferroni post-hoc test. For correlation analyzes, Pearson coefficient was used. *p* < 0.05 was determined to be statistically significant.

## Results

### Overexpression of circPARD3B inhibited VEGF production in the co-culture of OA-FLS and HDMECs and VEGF-induced angiogenesis

**FIGURE 1:**
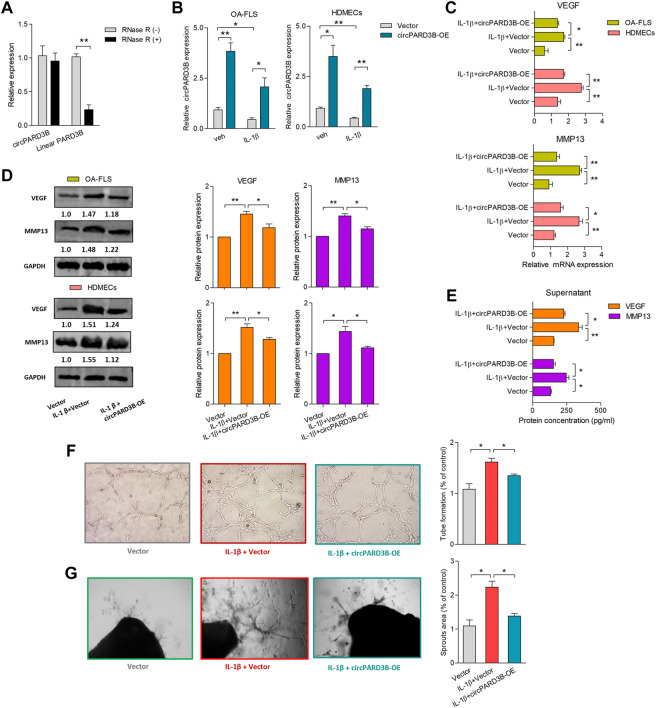
Overexpression of circPARD3B inhibited the production of VEGF and MMP13. mRNA **(A–C)** and protein **(D)** expression were respectively determined by qPCR and western blot analysis. **(A)** CircPARD3B’s resistance to Rnase R digestion (n = 3). **(B)** CircPARD3B’s expression in OA-FLS or HDMECs could be significantly downregulated by IL-1β induction, and then significantly elevated by circPARD3B overexpression (n = 3). **(C–E)** Role of circPARD3B overexpression in VEGF/MMP13 mRNA **(C)** as well as protein **(D)** expression in the OA-FLS or HDMECs co-culture, the concentration of VEGF in the supernatants of the co-culture **(E)** was significantly upregulated after induced by IL-1β, and then significantly downregulated *via* circPARD3B overexpression (n = 3). **(F–G)** Tube formation test **(F)**and *ex vivo* ARA of angiogenesis **(G)** proved the changes in the formation of capillary-like structure of HDMECs as well as microvessel sprouting with statistical significance based on the expression of VEGF in the supernatants of the OA-FLS and HDMECs co-culture (n = 3). Results are decribed with mean ± S.E.M. Veh = vehicle control. IL-1β = 10 ng/ml *p < 0.05, **p < 0.01.

Significantly up- or downregulated circRNAs in OA-FLS from the synovial tissues of knee joints had been reported (Xiang et al., 2019). Three of the most up-regulated circRNAs, along with three significantly down-regulated circRNAs, were validated by RT-qPCR analysis (Supplementary Figures S1A–C). To investigate the effects of these differentially expressed circRNAs on the pathogenesis of OA, experiments with overexpression or knockdown of circRNAs were carefully performed. However, the expression of their linear forms was not influenced with statistial significance (Supplementary Figures S2A,B, Figure 1A). Interestingly, VEGF and MMP13 mRNA expression was found to simultaneously be significantly down-regulated after overexpression of hsa_circ_0008172 (circPARD3B) (Supplementary Figures S2C,D); therefore, this circRNA was speculated to play a protective role and was selected to be further studied. Overexpression efficiency of circPARD3B in OA-FLS and SMSCs were evaluated by qPCR. The expression of PARD3B mRNA exihibited no significant alterations in response to circPARD3B overexpression in OA-FLS and SMSCs transfectants (Supplementary Figure S2E). As shown in Supplementary Figure S2F, circPARD3B that was overexpressed from the Adenovirus vectors were verified to resist RNase R’s digestion, while the linear forms were digested by RNase R. The coculture of OA-FLS and HDMECs co-culture was structured in the transwell system with IL-1β. Additionally, circPARD3B expression levels were down-regulated in OA-FLS and HDMECs coculture after IL-1β induction with statistical significance compared to those of the control group. Furthermore, this down-regulation could be clearly elevated by overexpressed circPARD3B (Figure 1B). VEGF and MMP13 gene transcription levels in co-cultured OA-FLS and HDMECs changed almost oppositely compared to those of circPARD3B. Namely, VEGF or MMP13 mRNA expression levels were significantly increased in OA-FLS/HDMECs under IL-1β induction, further respectively downregulated by circPARD3B overexpression (Figure 1C). Similar changes in VEGF/MMP13 protein expressions were found in the RA-FLS and HDMECs coculture plus protein secretion in the supernatants of the OA-FLS and HDMECs coculture, consistent with the level of mRNA expression (Figures 1D,E). Next, an *in vitro* TFA and *ex vivo* mouse aortic rings assay was performed under the transfer of supernatants from the coculture to HDMECs. And the changing trends between vascularity and the expression levels of VEGF in supernatants was found to be similar (Figures 1F,G). On the basis of the above, it could be inferred that overexpression of circPARD3B suppressed the angiogenesis induced by OA-FLS through the regulation of VEGF expression.

### CircPARD3B effects as a competing endogenous RNA for miR-326, further targeting SIRT1

**FIGURE 2:**
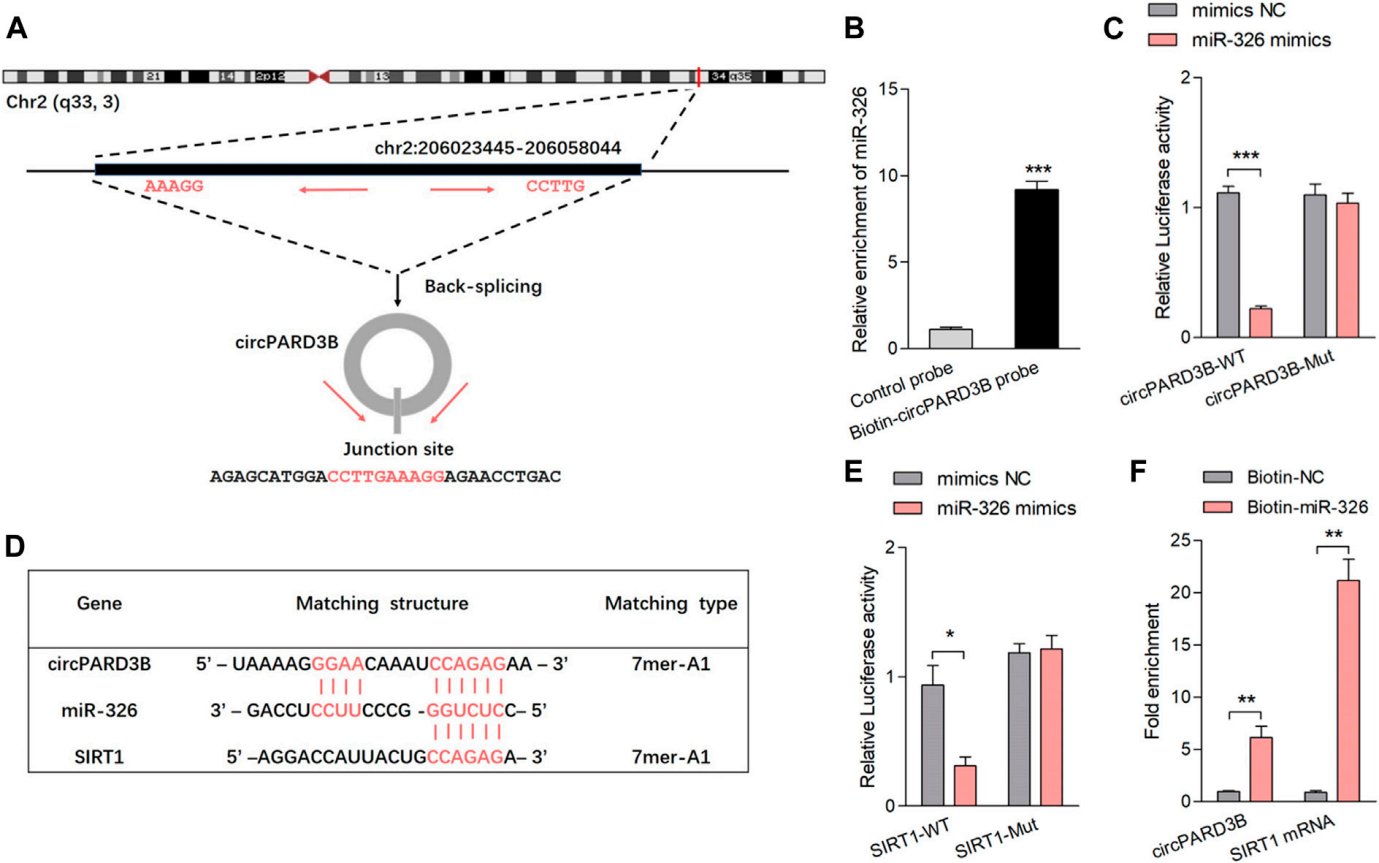
CircPARD3B interacted with miR-326, further targeting SIRT1. **(A)** Characteristics of hsa_circ_0008172 (circPARD3B). **(B)** MiR-326 was pulled down and enriched with biotinylated circPARD3B probe (n = 3). **(C)** When co-transfected with wild circPARD3B as well as miR-326 mimics, the relative luc activity of circPARD3B was evidently suppressed (n = 3). **(D)** Schematic graph illustrating binding sites between circPARD3B and miR-326, miR-326 and SIRT1 mRNA. **(E)** LRA revealed that the luc activitiy of SIRT1 wild type reporter was decreased with statistical significance when transfected with miR-326 mimics in contrast with control reporter or mutated luciferase reporter (n = 3). **(F)** An enrichment of circPARD3B as well as SIRT1 mRNA in the pulled down sediments of 3′ end biotin-labeled miR-326 with statistical significance (n = 3). Results are described with mean ± S.E.M. CTL = control group. Scrl-KD = scramble siRNA knockdown. NC = negative control. *p < 0.05, **p < 0.01, ***p < 0.001.

Characteristics of hsa_circ_0008172 (circPARD3B) were scanned in circBase (http://www.circbase.org/) (Figure 2A and Supplementary Figure S1D). Linear and circular RNA expressed in OA-FLS were determined by qPCR, and no significant difference of expression level between linear PARD3B and circPARD3B were observed (Supplementary Figure S1E). The location of CircPARD3B was shown to be mainly in the OA-FLS’s cytoplasm by FISH analysis (Supplementary Figure S3A), indicating that circPARD3B could function as a competing endogenous RNA to interact with miRNA. FISH results also showed that circPARD3B transcript signals in OA-FLS which overexpressed circPARD3B were mostly located in the cytoplasm of OA-FLS (Supplementary Figure S3B). The CircPARD3B-specific probe was produced by complementary oligonucleotides to the backsplice junction of circPARD3B. Lysates prepared from OA-FLS transfected with vector, circPARD3B-ir or circPARD3B were subjected to RNA pull-down assays with a circPARD3B probe or an oligo control probe. As was shown in Supplementary Figure S3C, a biotin-labeled circPARD3B probe targeting the splicing site of circPARD3B was proved to be able to efficiently pull down circPARD3B, and the pull-down efficiency was significantly strengthened by circPARD3B overexpression. CircPARD3B was predicted to probably interact with five miRNAs (miR-145, miR-326, miR-330-5p, miR-431, and miR-558) using bioinformatics, and was further confirmed by RPD analysis methods (Supplementary Figure S4A). The CircPARD3B-specific probe resulted in a 9-fold enriched miR-326 in the sediments in contrast to the control probe (Figure 2B). According to the definition of Arraystar Property Algorithms, at least 1 miRNA binding site of circPARD3B with the five miRNAs was predicted based on the CircInteractome database (Supplementary Figure S4B, Figure 2D). A luc assay aimed at detecting the binding of the 5 miRNAs with circPARD3B found that the activity of the luc was only reduced in miR-326, reaching up to not less than 50% (Supplementary Figure S4C, Figure 2C). Supplementary Figure S2 showed the efficiency of circPARD3B knockdown or overexpression. Potential targets of miR-326 were studied on the basis of the bioinformatic databases of Targetscan as well as Starbase. Furthermore, a putative binding site that was a sequence complementary to the seed region of miR-326 was predicted in the 3′UTR of SIRT1 mRNA (Figure 2D), and LRA demonstrated that when transfected with mimics of miR-326, the activation of the luc in the wild-type reporter of SIRT1 was reduced with statistical significance in contrast to the control reporter or the mutated reporter of luc (Figure 2E). The binding sites of circPARD3B with miRNAs including miR-326 and SIRT1 with miR-326 are shown in Supplementary Figure S5A,B, respectively. Furthermore, a significant enrichment of circPARD3B as well as SIRT1 mRNA by 3′end biotin-labelled miR-326 in the pulled down products confirms the results (Figure 2F, Supplementary Figure S4D).

### CircPARD3B inhibits VEGF and MMP13 expression *via* the miR-326/SIRT1 axis

**FIGURE 3:**
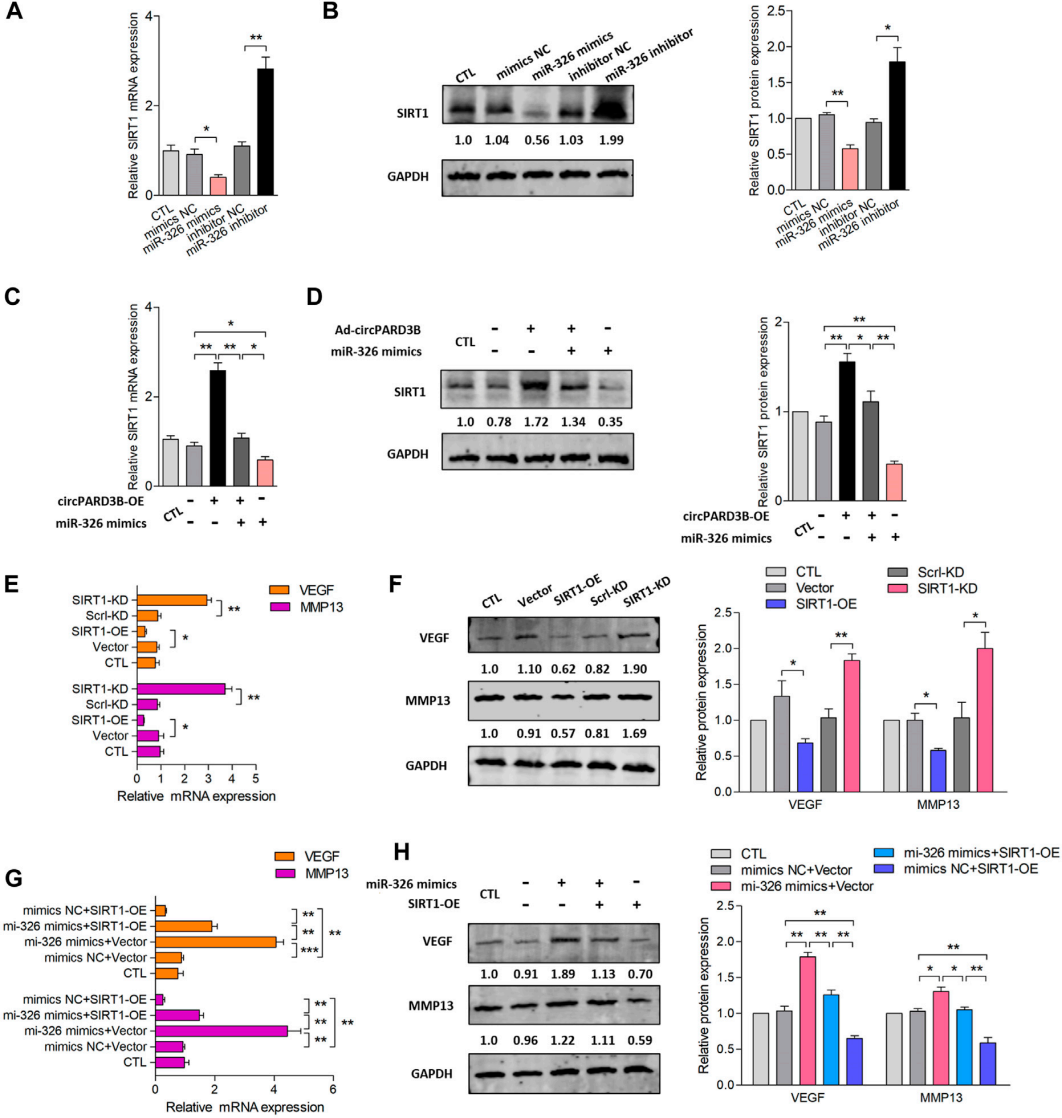
Inhibition of VEGF/MMP13 expression *via* circPARD3B/miR-326/SIRT1 axis *in vitro*. mRNA and protein expression were respectively determined by qPCR and western blot analysis. **(A,B)** SIRT1 mRNA **(A)** as well as protein expression **(B)** were down- or up-regulated by miR-326 mimics or inhibitor with statistical significance (n = 3). **(C,D)** Co-effects of circPARD3B and miR-326 on the expression of SIRT1 in OA-FLS were assessed by the detection of SIRT1 mRNA **(C)** as well as protein **(D)** expression. SIRT1 expression were elevated or reduced by circPARD3B overexpression or miR-326 mimics with statistical significance in contrast to each control group. Besides, the downregulation of SIRT1 expression suppressed by miR-326 mimics was able to be evidently recovered by overexpressed circPARD3B, which means that the upregulated expression of SIRT1 induced by overexpressed circPARD3B was able to be inhibited by miR-326 mimics with statistical significance (n = 3). **(E,F)** The mRNA **(E)** as well as protein **(F)** expression of VEGF/MMP13 in OA-FLS were reduced or elevated by SIRT1 overexpression or knockdown with statistical significance (n = 3). **(G,H)** MiR-326 mimics upregulated VEGF/MMP13 mRNA **(G)** as well as protein **(H)** expression, and these increasement was able to be suppressed by SIRT1 overexpression with statistical significance (n = 3). Results are described with mean ± S.E.M. CTL = PBS control group. Scrl-KD = scramble siRNA knockdown. NC = negative control. *p < 0.05, **p < 0.01.

SIRT1 mRNA and protein expression were negatively regulated by miR-326 mimics, but positively regulated by miR-326 inhibitor with statistical significance (Figures 3A,B). Furthermore, the down-regulation of SIRT1 level induced by miR-326 mimics could be recovered by overexpression of circPARD3B with statistical significance, suggesting that the up-regulation of SIRT1 expression carried out by overexpressed circPARD3B could be inhibited by miR-326 mimics with statistical significance (Figures 3C,D). On the basis of the above, it could be inferred that circPARD3B was able to regulate SIRT1 expression through miR-326.

Subsequently, the relationship between VEGF/MMP13 expression and circPARD3B/miR-326/SIRT1 axis was explored. First, we studied SIRT1’s effect on the expression of VEGF/MMP13. SIRT1 overexpression and silencing were established among OA-FLS. As shown in Supplementary Figure S6, the transfection efficiency of SIRT1 was stable. Figures 3E,F demonstrated that the overexpression of SIRT1 suppressed the expression of VEGF/MMP13, and the silencing of SIRT1 showed an enhanced influence on the levels of VEGF/MMP13. In particular, the up-regulated expression of VEGF/MMP13 induced by SIRT1 overexpressed was able to be inhibited by miR-326 mimics; besides, VEGF/MMP13 expression could also be significantly reduced by miR-326 mimics (Figures 3G,H). Based on these findings, it could be inferred that SIRT1 inhibited the expression of VEGF/MMP13 between OA-FLS, indicating that VEGF/MMP13 could be downstream of the circPARD3B/miR-326/SIRT1 axis.

### Construction of OE-circPARD3B-SMSCs-sEV and evaluation of its antiangiogenic effect on the coculture of OA-FLS and HDMECs

**FIGURE 4:**
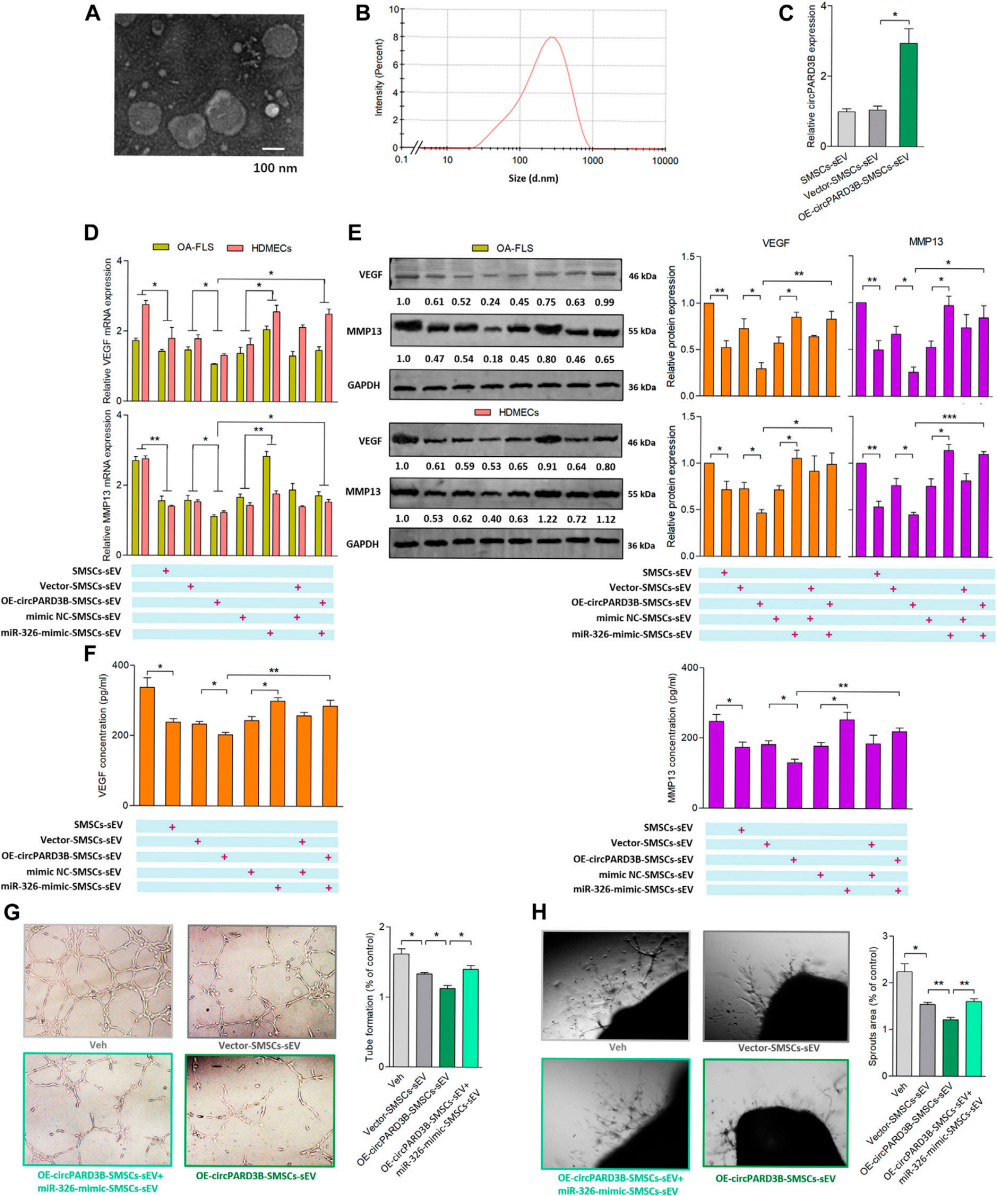
Evaluation of OE-circPARD3B-SMSCs-sEV’s anti-angiogenic effect on OA-FLS & HDMECs co-culture. SMSCs-sEV were confirmed by TEM **(A)** as well as DLS **(B)** as an average diameter of approximately 100 nm. mRNA **(C,D)** and protein **(E)** expression were respectively determined by qPCR and western blot analysis. **(C)** OE-circPARD3B-SMSCs-sEV carried enhanced expression of circPARD3B in contrast with those from Vector-SMSCs-sEV with statistical significance (n = 3). Under incubation of IL-1β, VEGF/MMP13 mRNA **(D)**, as well as protein **(E)** expression in the OA-FLS and HDMECs co-culture and protein in the supernatants of the OA-FLS and HDMECs co-culture **(F)** were found to be downregulated by OE-circPARD3B-SMSCs-sEV, which was able to be inhibited by miR-326-mimic-SMSCs-sEV with statistical significance (n = 3). Changing trends of capillary-like structure formation in tube formation assay **(G)** as well as microvessel sprouting in ARA of angiogenesis **(H)** were in consistency with the changes of the expression of VEGF in supernatants (n = 3). Results are described with mean ± S.E.M. Veh = vehicle control *p < 0.05, **p < 0.01.

The therapeutic effect of SMSCs-sEV on synovial angiogenesis was confirmed in our previous studies, and we assumed that circPARD3B was able to move into target cells as a cargo inside SMSCs-sEV. Therefore, SMSCs stably overexpressing circPARD3B (OE-circPARD3B-SMSCs) were constructed after infected with the adenovirus; In addition, sEV secreted by OE-circPARD3B-SMSCs were prepared for further experiments. The verification of SMSCs-sEV was demonstrated based on TEM (Figure 4A), DLS detection (Figure 4B), and WB analysis (Supplementary Figure S7A). Furthermore, circPARD3B-OE, miR-326 mimics, as well as their corresponding negative controls (Vector/mimics NC) were transfected into SMSCs. In addition, to deliver circPARD3B (OE-circPARD3B-SMSCs-sEV) or miR-326 (miR-326-mimic-SMSCs-sEV), sEV were separated from the culture medium. The efficiency of overexpression of circPARD3B in SMSCs is shown in Supplementary Figure S7B. CircPARD3B expression in OE-circPARD3B-SMSCs-sEV, miR-326 level in miR-326-mimic-SMSCs-sEV were respectively confirmed by qRT–PCR (Figure 4C, Supplementary Figure S7C). To investigate the effects of OE-circPARD3B-SMSCs-sEV on angiogenesis in the OA synovium, SMSCs-sEV, OE-circPARD3B-SMSCs-sEV, and miR-326-mimic-SMSCs-sEV were added to the OA-FLS and HDMECs coculture, followed by incubation with IL-1β for 48 h. Then VEGF/MMP13 mRNA (Figure 4D) and protein expression (Figure 4E) in the coculture of OA-FLS and HDMECs were found to be down-regulated by SMSCs-sEV with statistical significance, more significantly down-regulated by OE-circPARD3B-SMSCs-sEV. However, this down-regulation was able to be inhibited by miR-326-mimic-SMSCs-sEV, which could significantly up-regulate VEGF/MMP13 expression by itself (Figures 4D,E). The changing trends of the concentration of VEGF/MMP13 in the supernatants obtained from the OA-FLS and HDMECs co-culture (Figure 4F), plus their induced vascular structures formed by HDMECs (Figures 4G,H) were found to be similar, indicating that the angiogenic activity induced by the OA-FLS and HDMECs coculture was significantly suppressed by OE-circPARD3B-SMSCs-sEV. The fusion of SMSCs-sEV labeled with PKH67 and OA-FLS/HDMECs was determined by fluorescence microscopy (Supplementary Figure S7D).

### Serum-derived sEV circPARD3B as a potential biomarker for osteoarthritis phenotypes

**FIGURE 5:**
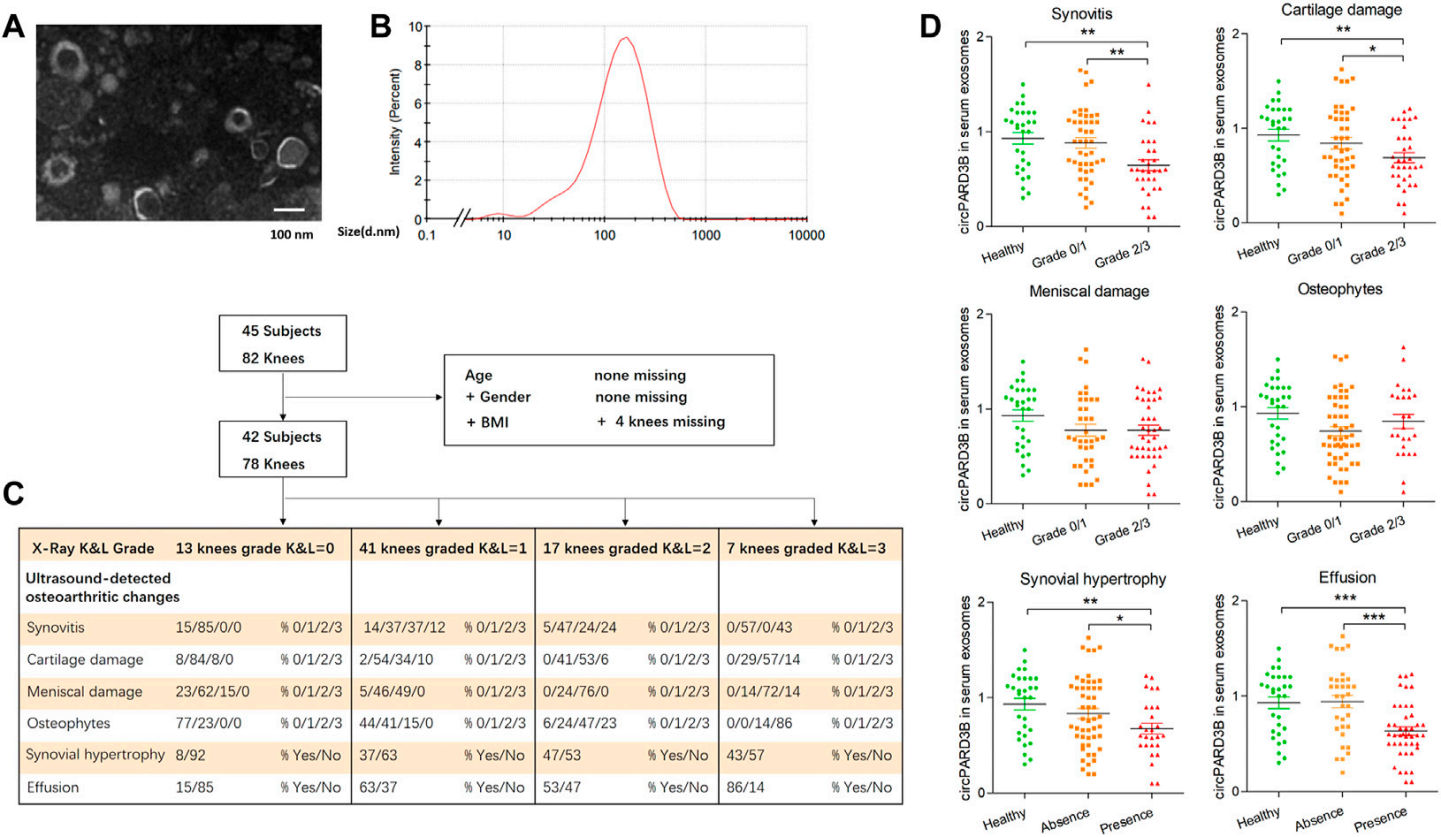
Serum-derived sEV circPARD3B as a potential biomarker for inflammatory phenotype of knee OA. Serum sEV were confirmed by TEM **(A)** and DLS **(B)**. **(C)** Characteristic of OA patients under ultrasonography grading. **(D)** CircPARD3B levels in serum sEV were determined by qPCR. Correlations between serum sEV circPARD3B levels and sonographical grading characteristics of knee OA patients. Results are described with mean ± S.E.M *p < 0.05, **p < 0.01, ***p < 0.001.

Since circPARD3B was verified to play a critical role in the angiogenic event under inflammation, expressed by OA-FLS synovial key cells and endothelial cells, in addition to the detection of circPARD3B in sEV derived from SMSCs, we are curious whether circPARD3B was present in serum sEV from OA patients, representing a valuable tool to identify the special phenotype of patients with OA in which synovial angiogenesis secondary to local inflammation play a key role in disease progression. Healthy controls and patients with OA were selected and serum sEV were extracted and confirmed (Figures 5A,B). The clinical and sonographic characteristics of the patients were demonstrated in Supplementary Table S2 and Figure 5C. As illustrated in Supplementary Figures S8A,B, there was no significant difference in serum sEV circPARD3B levels between OA patients and non-OA controls, as well as in patients with different grades of KL defined under X-ray detection. And no/weak correlation was observed between CRP/ESR levels with serum sEV-derived circPARD3B quantified by RT-qPCR (Supplementary Table S3). Interestingly, under ultrasound detection, sEV- linked circPARD3B levels in serum of OA patients with synovitis grade 2 or 3 were significantly lower than in patients with synovitis grade 0 or 1, as well as healthy controls (Figure 5D). As ultrasound synovitis often indicates local inflammation, these data suggested that serum sEV circPARD3B could serve as a novel biomarker for the inflammatory phenotype of OA.

### OE-circPARD3B-SMSCs-sEV suppressed OA in the OA mice model induced by collagenase

**FIGURE 6:**
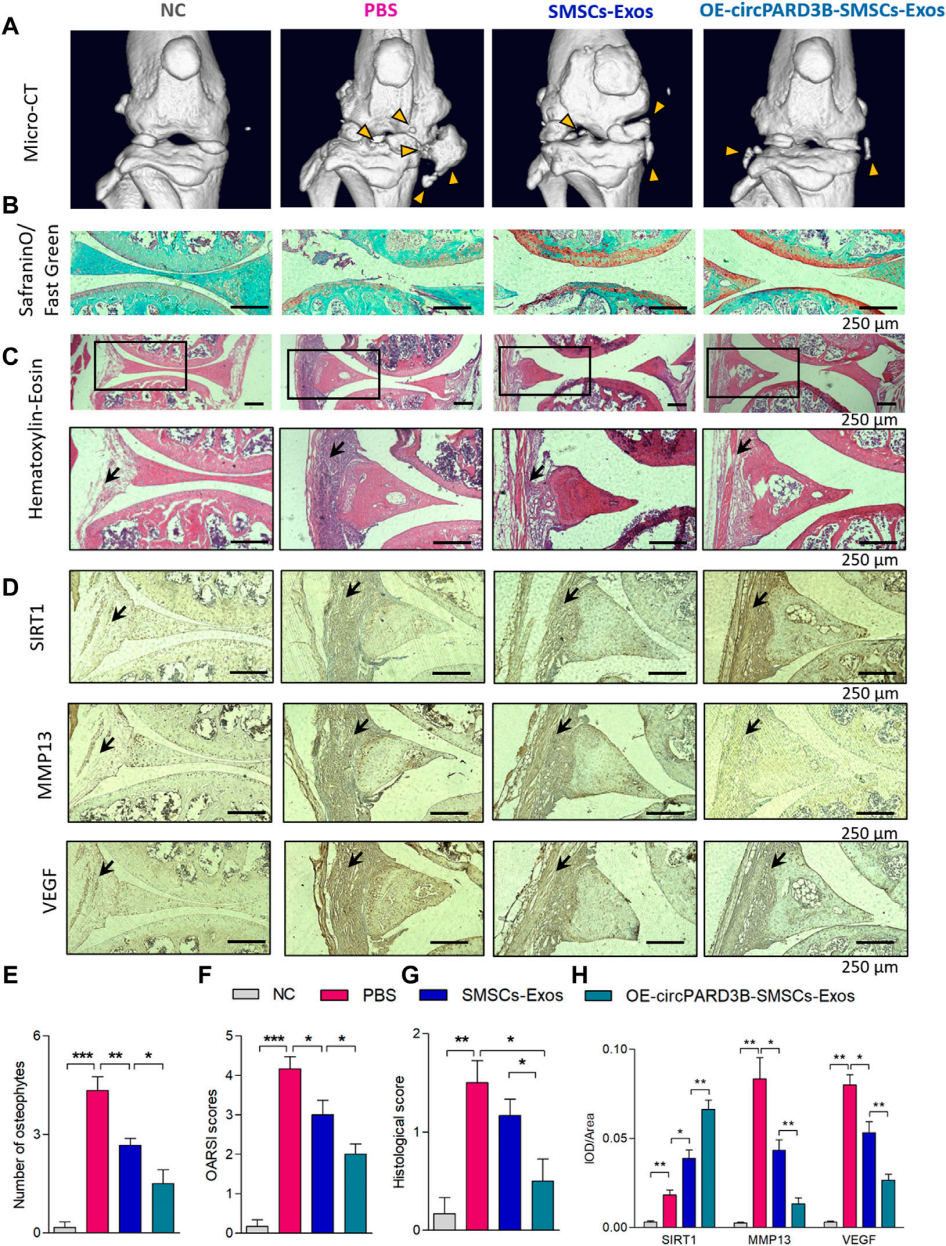
Injection of OE-circPARD3B-SMSCs-sEV alleviate the progression of arthritis in collagenase-induced OA mouse model. **(A)** Representative micro-CT images of knee joints showing the abnormal growth of osteophytes (yellow arrow). **(B)** Representative images of Safranin O/Fast Green staining of knee cartilage from mice. **(C)** Representative images of HE staining analysis of knee joints from mice. After induced by OA, mice exhibited the mild inflammatory infiltration along with the synovial hyperplasia in the synovial tissues. Joint inflammation was reduced in the SMSCs-sEV group in contrast to the OA control group with statistical significance, especially in OE-circPARD3B-SMSCs-sEV group in contrast to BMSCs-sEV group. **(D)** IHC staining of SIRT1, VEGF and MMP13 were respectively demonstrated. We observed strengthened VEGF/MMP13 staining in synovial tissues of the OA control group in contrast to NC mice. OE-circPARD3B-SMSCs-sEV or SMSCs-sEV treated OA group revealed stregthened SIRT1 and weakened VEGF/MMP13 staining intensity in synovial tissues in contrast to the OA control mice, especially obvious in OE-circPARD3B-SMSCs-sEV group in contrast to that in the SMSCs-sEV group. **(E)** Quantification of images with 3D reconstruction by counting the number of osteophytes. **(F)** OARSI scoring to assess the histological changes of knee cartilage. **(G)** Histological assessment scores were shown. **(H)** Integrated optical density (IOD) values of immunostaining were shown in average. Scale bars = 250 µm. Data are described with mean ± S.E.M. n = 6; *p < 0.05, **p < 0.01, ***p < 0.001. NC = PBS treated normal control mice. OA control group = PBS treated OA group.

To further investigate the influence of OE-circPARD3B-SMSCs-sEV on OA *in vivo, collagenase-induced* OA mice were administered with OE-circPARD3B-SMSCs-sEV, Vector-SMSCs-sEV, as well as SMSCs-sEV twice a week by intraarticular injection. Knee joints were harvested from four groups: NC without collagenase injection, OA control, SMSCs-sEV, Vector-SMSCs-sEV as well as OE-circPARD3B-SMSCs-sEV. After the first injection of collagenase type VII, treatment was carried out continuously from day 7 to day 42. The 3D reconstruction of the microCT images taken on day 42 demonstrated an increase in osteophytes in knee joints of OA mice, while SMSCs-sEV intraarticular injection decreased osteophytes, and the inhibitory effect was even better in the OE-circPARD3B-SMSCs-sEV treatment group (Figure 6A). The number of osteophytes was also evaluated in different groups (Figure 6E), and there was no significant difference in osteophyte formation in the SMSCs-sEV treatment and the Vector-SMSCs-sEV treatment (Supplementary Figure S9A).

Safranin O and Fast Green staining (Figure 6B) revealed that cartilage surfaces in collagenase-induced OA mice improved after injection of OE-circPARD3B-SMSCs-sEV or SMSCs-sEV, and the improvement was even more evident in the OE-circPARD3B-SMSCs-sEV group. Quantitative analysis with the Osteoarthritis Research Society International (OARSI) scoring was consistent with the Safranin O and Fast Green staining, showing that OE-circPARD3B-SMSCs-sEV or SMSCs-sEV significantly reduced OARSI scores (Figure 6F), and no evident differences were observed in SMSCs-sEV treatment compared to Vector-SMSCs-sEV treatment (Supplementary Figure S9B).

Histopathological evaluation of H&E stained sections stained by H&E showed that after induction of OA, to some extent, mice presented inflammatory infiltration as well as synovial hyperplasia in synovial tissue; compared to the control group administered with PBS, bone and cartilage were obviously much less destructed in the SMSCs-sEV group as well as the OE-circPARD3B-SMSCs-sEV group. Furthermore, the pathological changes in the joints in the OE-circPARD3B-SMSCs-sEV group were much better compared to those in the SMSCs-sEV group (Figures 6C,G).

IHC staining of the sections of joint tissues and the mean IOD showed an increased intensity of SIRT1 and VEGF staining, aggravated degenerative effects in the cartilage matrix, such as expression of the enzyme MMP13 degrading the ECM in control mice with OA in contrast to NC mice. Furthermore, in contrast to the OA control group, SMSCs-sEV strengthened the intensity of SIRT1 staining but weakened VEGF and MMP13. Furthermore, compared to the SMSCs-sEV group, the OE-circPARD3B-SMSCs-sEV group showed stronger IHC staining of SIRT1 but weaker staining of VEGF/MMP13 (Figures 6D,H). No obvious differences in H&E or IHC staining were found in the SMSCs-sEV group or in the treatment with Vector-SMSCs-sEV (Supplementary Figures S9C,D). As VEGF was verified to exert a key effect on synovial angiogenesis in our previous study (Zhang et al., 2017), the elevated circPARD3B through SMSCs-sEV transfer exerted a positive effect on inhibition of angiogenesis plus ECM catabolism to alleviate OA *in vivo*.

## Discussion

More than 1000 sEV-derived circRNAs have already been found in the serum of human beings (Fanale et al., 2018). Benefiting from easier detection in peripheral blood, serum sEV circRNAs have been recognized as ideal biomarkers for the diagnosis of human diseases considering the advantages of predominant expression, conservative evolution, high resistance to RNase, as well as specificity for tissues and stages (Fanale et al., 2018) In this study, it was found for the first time that serum sEV circPARD3B could be a biomarker with a significantly reduced level in some osteoarthritis patients, unlike other sEV-derived circRNAs reported as biomarkers that were usually increased in serum (Wang et al., 2019; Ding et al., 2020). More importantly, we first applied this serum sEV circPARD3B to distinguish a OA phenotype and found that this circPARD3B exerted a pivotal effect on the pathogenesis of this subtype, successfully grouping patients with knee OA into different phenotypes, i.e., serum sEV circPARD3B combined with synovial inflammation in knee joints. Serum sEV-linked circRNAs have always been reported to be significantly up-regulated due to the mechanism of promoting cancer growth by delivering pathogenic circRNAs to the cancer focus *via* serum sEV (Ding et al., 2020; Lu et al., 2020), and quite rightly, it is not yet clear whether the decreased sEV circPARD3B will also be transferred to other joints or even organs, and then affect the progression of OA or not, worthy of our future study.

Obviously, different from previous studies that only screened biomarkers in OA (Zhang et al., 2018) or only explored circRNAs as therapeutic targets (Shen et al., 2019; Shen et al., 2021), a more striking advantage of this study is that we found that circPARD3B not only decreased serum sEV, but also exerted a critical pathogenic effect on the progression of OA, probably a therapeutic target; thus specific genetically modified sEV were constructed for circRNA-targeted treatment. After all, circRNA, as a highly tissue and stage-specific molecule, exerts a pivotal inflence on the onset and development of diseases with a high degree of heterogeneity (Kristensen et al., 2019). For example, there have been many reports in the literature on the effect of circRNAs on the pathogenesis of OA (Li et al., 2018; Shen et al., 2019; Shen et al., 2021). Making full use of the heterogeneity of circRNAs will be of great significance. The axis of alteration of the serum sEV-linked circRNA expression level, thus identifying of the phenotypes, then infusion therapy of the corresponding circRNA modified sEV would provide an important strategy not only for OA, but also for other diseases.

In addition, the advantage of this study is that for the first time we revealed the pivotal influence of circRNAs on the angiogenic event of OA. This is different from the other published reports which mostly focused on the role of circRNAs as important regulators of proliferation, apoptosis, or autophagy of chondrocytes (Mao et al., 2021). OA is related to articular cartilage loss, synovial inflammation, fibrosis, subchondral bone remodeling, and osteophyte formation, each of which may be attributed to the angiogenesis (Bonnet and Walsh, 2005). Despite this, the relationship of circRNAs as well as the angiogenesis in OA remains undetermined. Therefore, the important effect of angiogenesis on OA pathogenesis has recently been of great concern. Revealing the changes and role of small molecular circRNAs is undoubtedly more conducive to the precise treatment of OA. In our study, 1 circRNA (hsa_circ_0008172, circPARD3B) was found to target miR-326, which could bind to SIRT1 and further inhibit downstream VEGF, thus exerting a pivoral effect on the angiogenic event. Initially considered cartilage-driven, OA is a much more complex disease with low-grade inflammation induced by the metabolic syndrome, innate immunity, and inflammation (Berenbaum, 2013). VEGF-induced angiogenesis induced by VEGF in the low-grade inflammation has been a great challenge for the treatment of diseases such as OA and others linked to inflammatory angiogenesis. To our knowledge, this circRNA is related to the angiogenesis of OA for the first time. Furthermore, in this study we showed that VEGF expression could be reduced by overexpressed SIRT1, indicating that VEGF could be the downstream mediator for SIRT1, which was consistent with previously reported results (Zhang et al., 2015; Karbasforooshan and Karimi, 2018). SIRT1 signaling has been associated with aging, caloric intake, and immune responses, and consistently, SIRT1 has also been associated with various age-associated diseases such as neurodegenerative diseases (Chen et al., 2020), type II diabetes (Karbasforooshan and Karimi, 2018), and immune-associated diseases (Yu et al., 2018). The functional module of circPARD3B/miR-326/SIRT1 might offer a new therapeutic target for other diseases among which SIRT1 exerts a pivotal effect even in its premature stages.

In this study, a special type of genetically modified sEV was constructed. sEV are extracellular vesicles secreted by most eukaryotic cells and participate in intercellular communication, with great potential as novel drug carriers (Batrakova and Kim, 2015). MSC-derived sEV (MSCs-sEV) have been shown to provide new perspectives for the development of cell-free MSCs therapy for cartilage injuries *via* possible action on cartilage repair in the context of the widely reported immunomodulatory and regenerative potency of MSCs-sEV (Kim et al., 2020). In this study, SMSCs-sEV were verified to inhibit angiogenesis under low-grade inflammation, according to the previous report (Chen et al., 2018)*.* Therefore, genetically modified OE-circPARD3B-SMSCs-sEV could deliver circPARD3B to recipient OA-FLS and endorhelial cells to inhibit angiogenesis, indicating the possibility of a new sEV therapy targeting circRNA. Furthermore, OE-circPARD3B-SMSCs-sEV might possibly have advantages over simple circPARD3B overexpression therapy. As sEV can also be transformed into more effective therapeutic drugs by simultaneously carrying other therapeutic non-coding RNAs targeting the SIRT1 gene (Cui et al., 2019) or small molecule drugs (Di Benedetto et al., 2021), which would benefit diseases with similar antiangiogenic effects.

The methodological limitations of this study shold be pointed out. Most assays were based on overexpression of circRNAs and reporters. Full control of the RNA form (length and circ-to-linear ratio) and amount could be allowed by transfection of in vitro-generated circRNAs, but endogenous circRNA function may not be recapitulated. Besides, both linear and circular forms are often produced by circRNA-expressing vectors (Nielsen et al., 2022). In this study, the levels of transfected circPARD3B plus its linear form has been monitored to ensure that effects attributed to circRNA are not driven by linear form.

**FIGURE 7:**
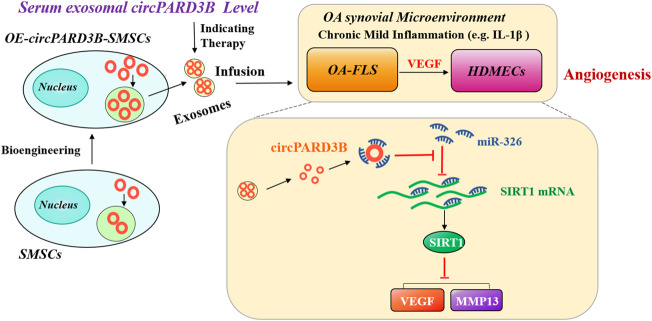
The mechanism of OE-circPARD3B-SMSCs-sEV therapy in OA. SMSCs stably overexpressing circPARD3B (OE-circPARD3B-SMSCs) were constructed after infected by the adenovirus. CircPARD3B derived from OE-circPARD3B-SMSCs might play a role as a sponge for the SIRT1-targeting miR-326. The downstream VEGF along with its conducted angiogenesis was then suppressed, with reduced expression of extracellular matrix (ECM) degrading enzyme MMP13 under local inflammatory microenvironment, such as IL-1β, ultimately inhibition of OA disease progression. Therefore, in contrast with the treatment of single SMSCs-sEV, the intracellular transfer of circPARD3B by OE-circPARD3B-SMSCs-sEV showed the better curative effect. Serum sEV circPARD3B level was found to be significantly downregulated in special phenotype OA patients with evident synovitis, suggesting that serum sEV circPARD3B might serve as a novel biomarker for the inflammatory phenotype of OA as well as an optimistic therapeutic effect of OE-circPARD3B-SMSCs-sEV towards this subgroup patients, indicating a potential novel therapeutic strategy for OA.

In conclusion, serum sEV circPARD3B is down-regulated in the inflammatory phenotype of osteoarthritis patients. CircPARD3B inhibits synovial angiogenesis by sponging miR-326 to up-regulate SIRT1 thus reducing the level of VEGF and MMP13 under local low-grade inflammation, which ultimately improved OA (Figure 7). Results of this study implies that sEV-linked circPARD3B is a new circulating biomarker for OA. Furthermore, inhibition of circPARD3B on angiogenesis in both effector cells OA-FLS and microvascular endothelial cells also indicates a novel target for OA treatment. In addition, circPARD3B-overexpressing SMSCs-sEV may play a role as key agents in the development of novel precision therapeutic treatments for OA.

## Data Availability

The raw data supporting the conclusions of this article will be made available by the authors, without undue reservation.

## Abbreviations

OA, osteoarthritis; circRNAs, circular RNAs; miRNAs, microRNAs; MSCs, mesenchymal stem cells; SMSCs, synovial mesenchymal stem cells; sEV, small Extracellular vesicles; SMSCs-sEV, SMSCs derived sEV; OE-circPARD3B-SMSCs, SMSCs transfected with circPARD3B expression adenovirus; OE-circPARD3B-SMSCs-sEV, sEV secreted from OE-circPARD3B-SMSCs; OA-FLS, fibroblast-like synoviocytes of OA patients; HDMECs, human dermal microvascular endothelial cells; VEGF, vascular endothelial growth factor; SIRT1, sirtuin 1; MMP13, matrix metalloproteinases 13; TEM, transmission electron microscope; DLS, Dynamic Light Scattering; FISH, fluorescence *in situ* hybridization; ARA, aortic ring assay; RPD, RNA pull-down; BT, biotinylated; LRA, Luciferase reporter assay; luc, luciferase; IOD, integrated optical density; FCM, flowcytometry; micro-CT, micro-computed tomography; H&E, hematoxylin-eosin.
