# Supplementary material for: Potential predictive and therapeutic applications of small extracellular vesicles-derived circPARD3B in osteoarthritis
Source: Front Pharmacol. 2022 Oct 19;13:968776. doi: 10.3389/fphar.2022.968776 (PMC9627215; doi:10.3389/fphar.2022.968776)

**Figure1F--Tube formation piture**

Vector IL-1bata+Vector IL-1bata+circRNA-OE


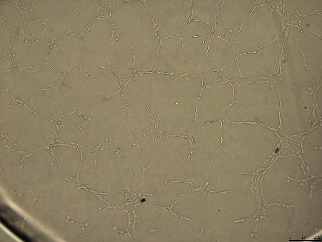

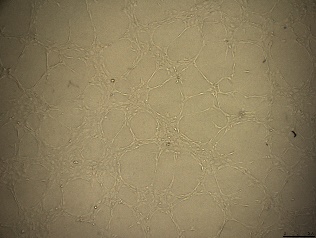

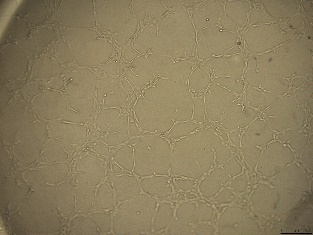


**Figure1G-Ring Picture**

IL-1bata+circRNA-OE Vector IL-1bata+Vector


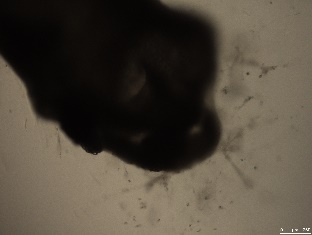

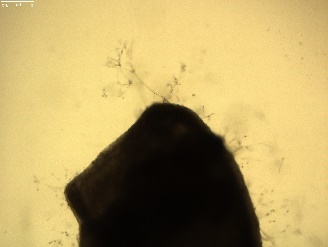

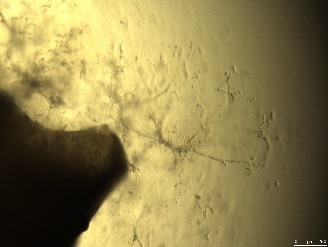


**Figure4**

Figure4A


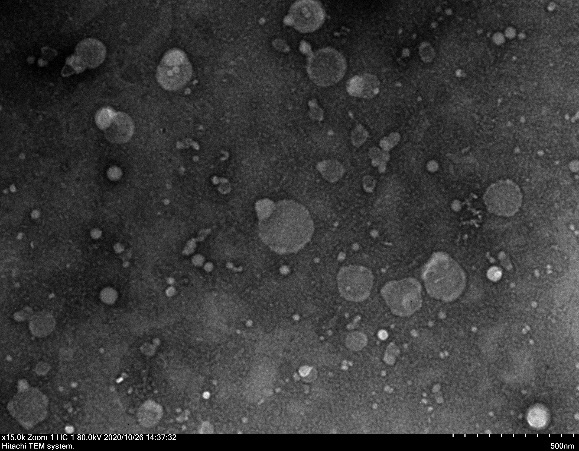


Figure4H

OE-circPARD3B-SMSCs-Exos OE-circPARD3B-SMSCs-Exos+miR326


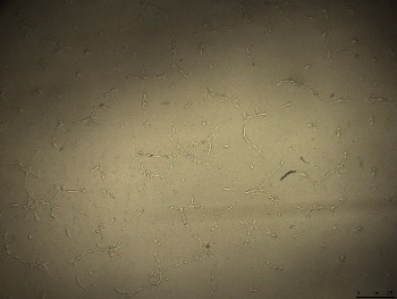

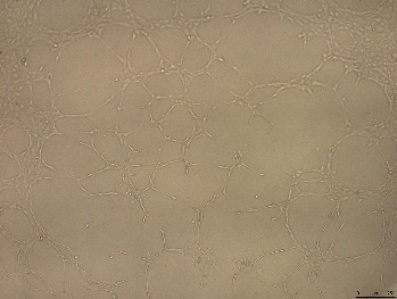


Vector-SMSCs-Exos Veh


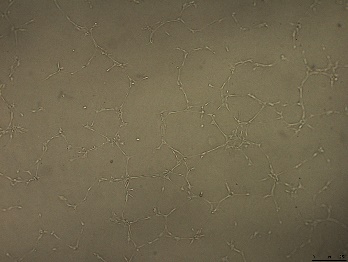

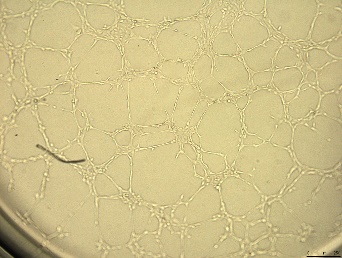


Figure4I

OE-circPARD3B-SMSCs-Exos OE-circPARD3B-SMSCs-Exos+miR-326


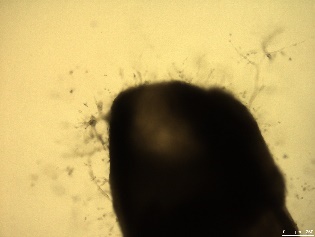

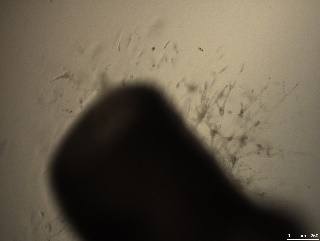


Vector-SMSCs-Exos Veh


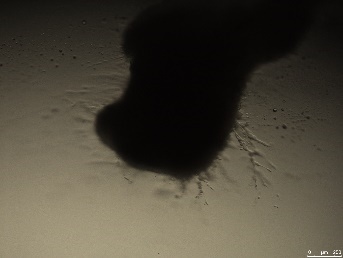

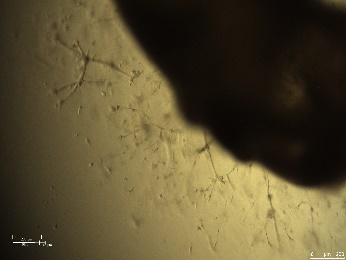


**Figure5A**


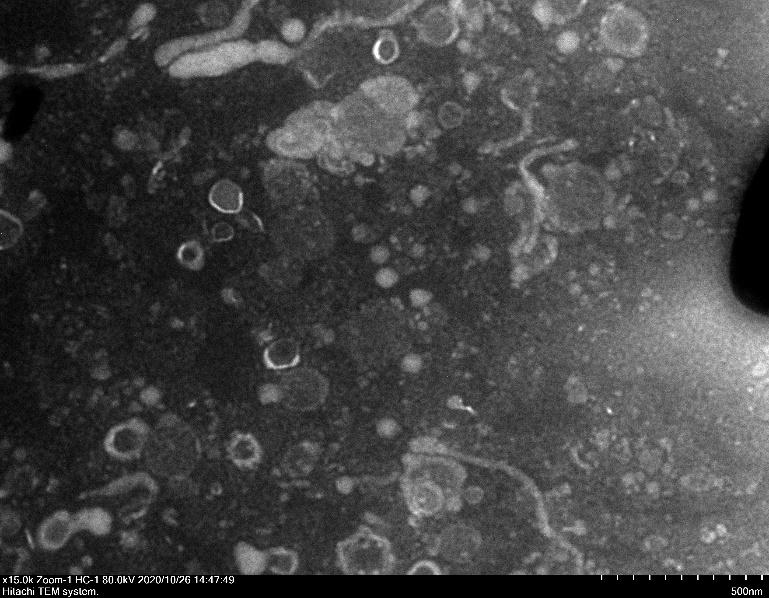


**Figure6**

Figure6A

NC OE-circPARD3B-SMSCs-Exos


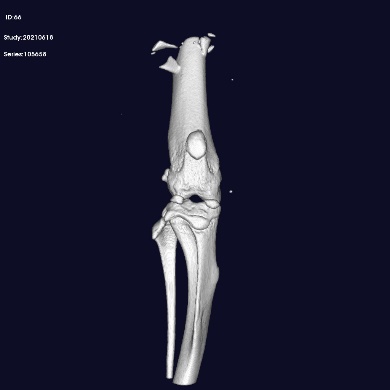

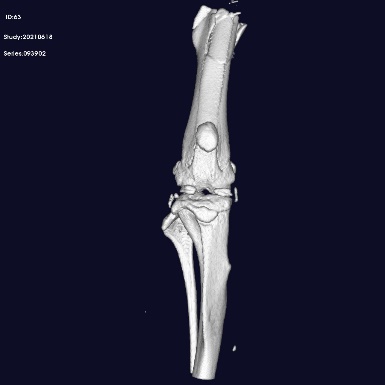


PBS SMSCs-Exos


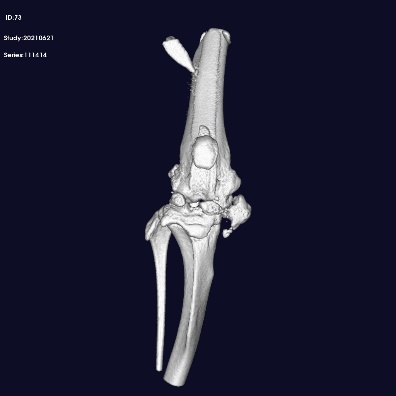

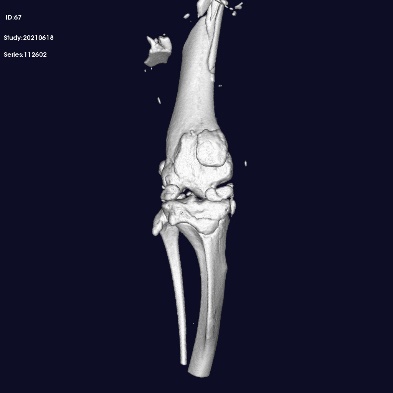


Figure6B

NC OE-circPARD3B-SMSCs-Exos


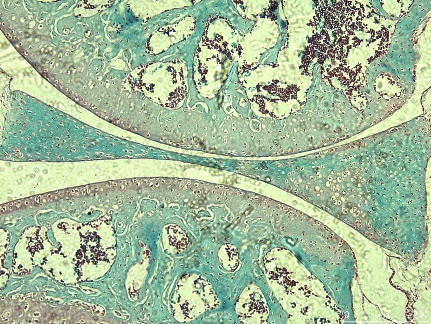

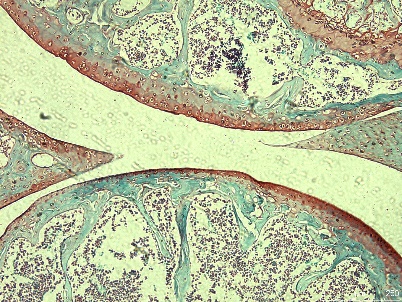


PBS SMSCs-Exos


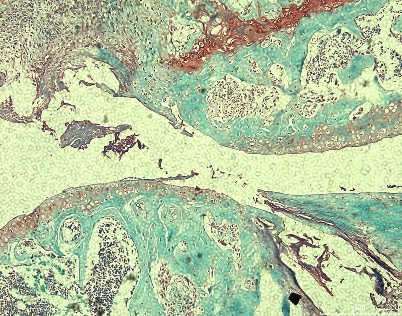

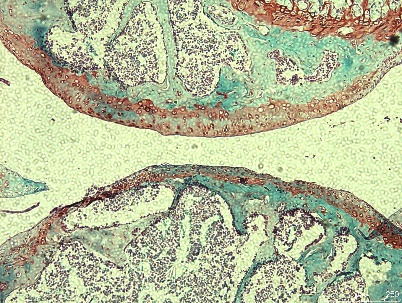


Figure6C

HE× 50 magnification

NC circPARD3B-SMSCs-Exos


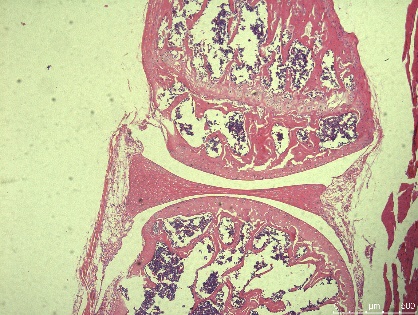

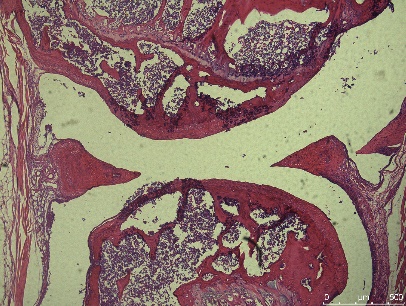


PBS OE-circPARD3B-SMSCs-Exos


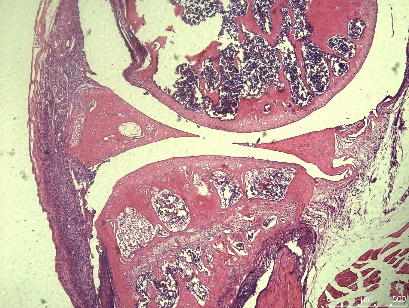

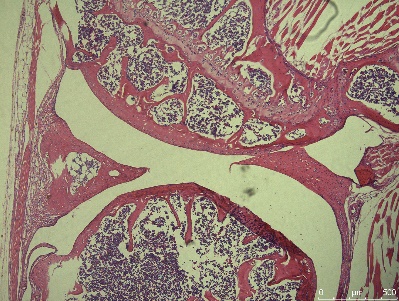


HE× 100 magnification

NC SMSCs-Exos


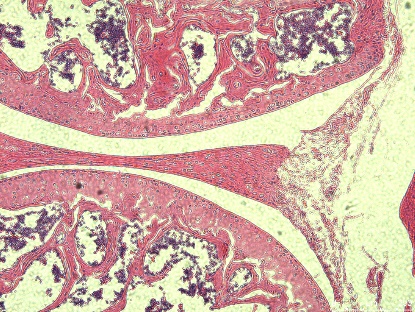

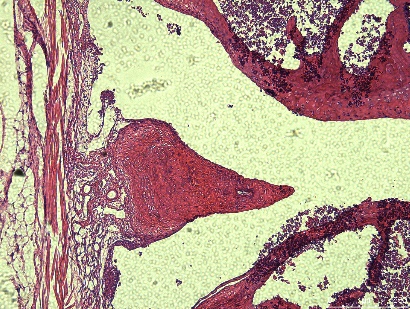


PBS OE-circPARD3B-SMSCs-Exos


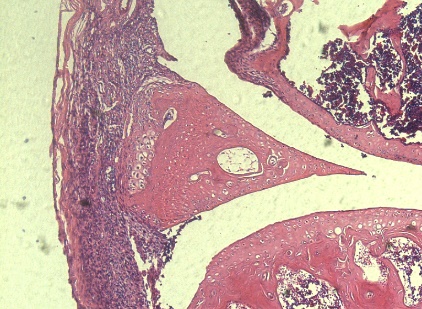

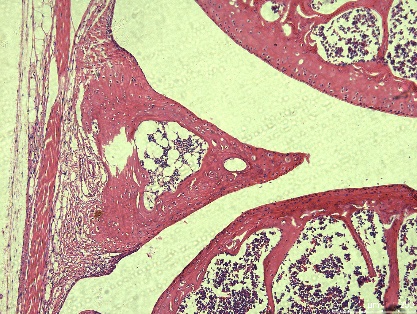


Figure6D

MMP13

NC SMSCs-Exo


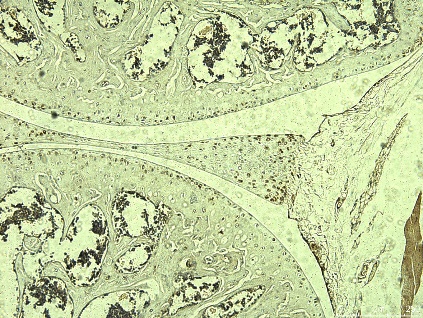

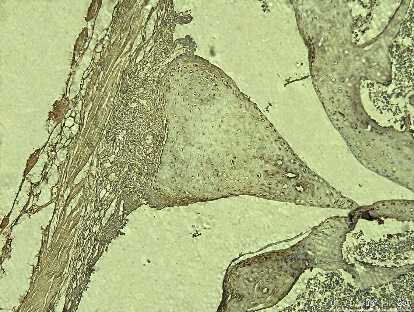


PBS OE-circPARD3B-SMSCs-Exos


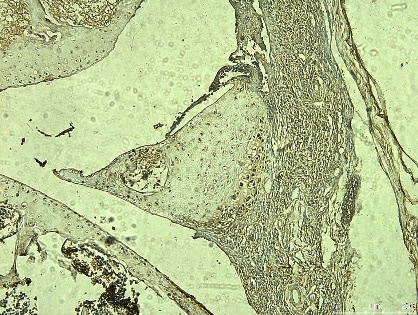

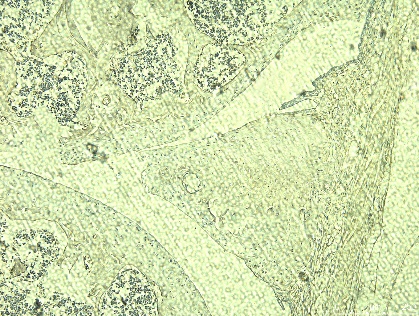


SIRT1

NC SMSCs-Exo


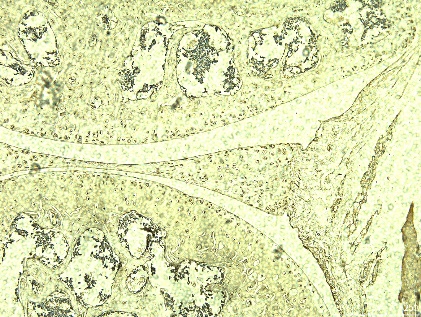

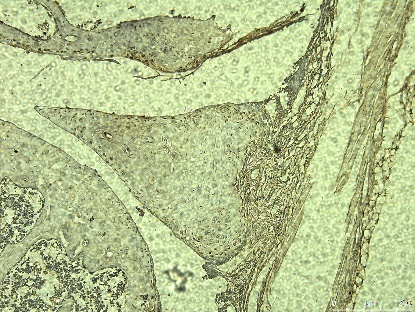


PBS OE-circPARD3B-SMSCs-Exos


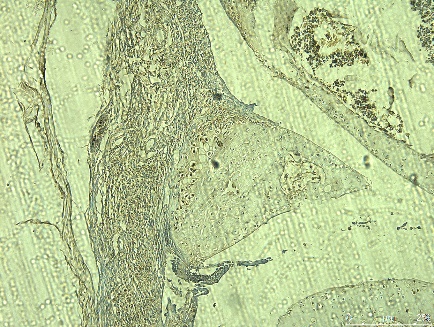

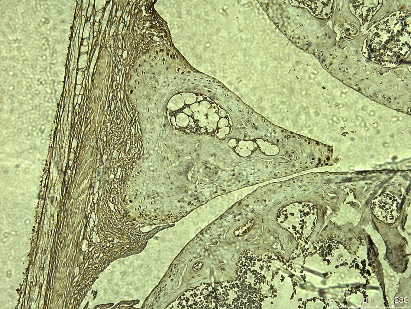


VEGF

NC SMSCs-Exo


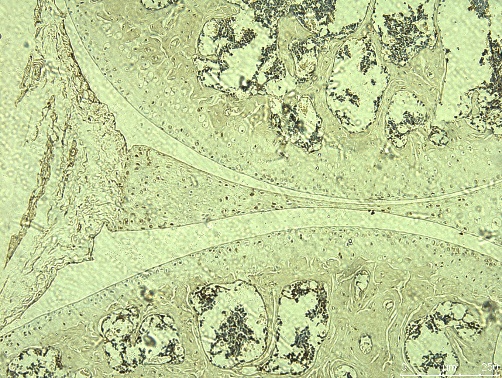

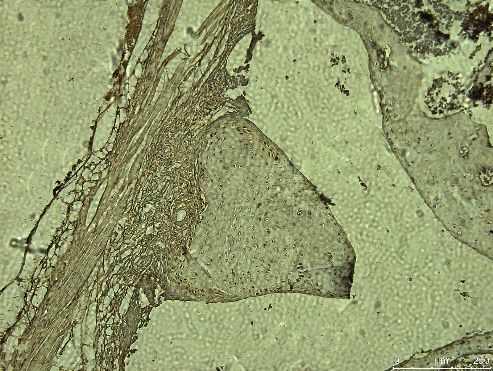


PBS OE-circPARD3B-SMSCs-Exos


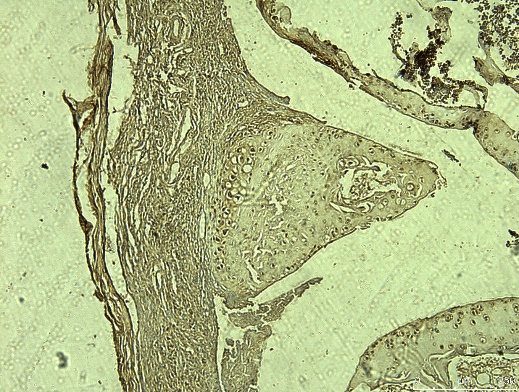

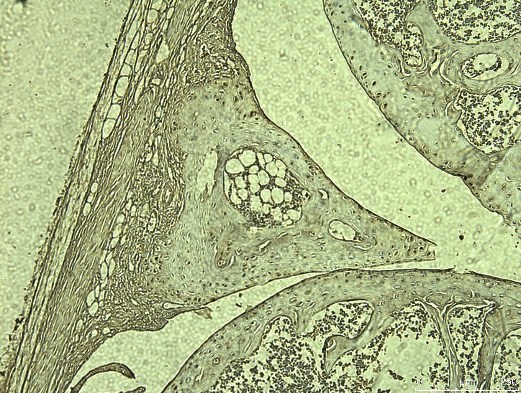


**Supplementary Figure 3**

**18s**

**Cy5 DAPI Merge**


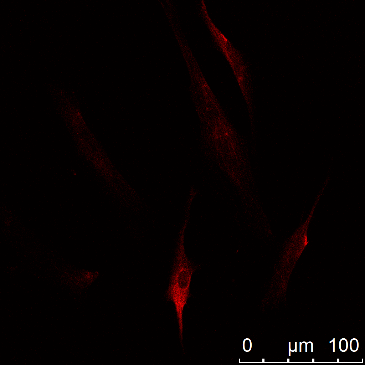

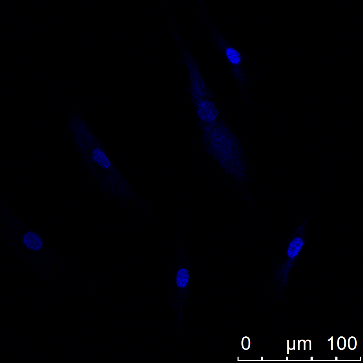

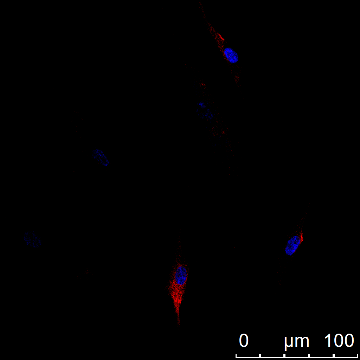


**circPARD3B**

**Cy5 DAPI Merge**


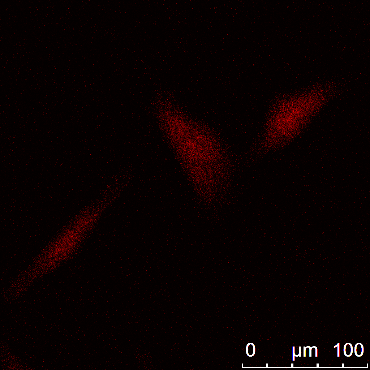

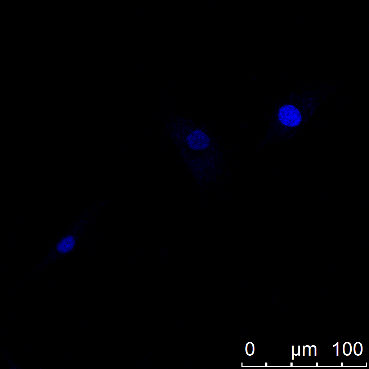

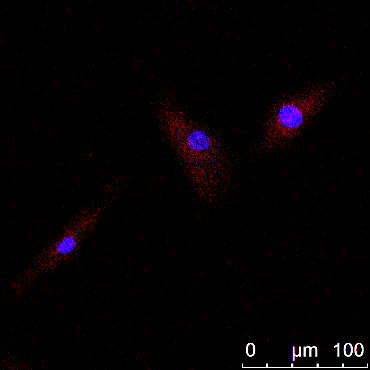


**Supplementary Figure 7**

**FLS**

**DAPI PKH67 overlay**


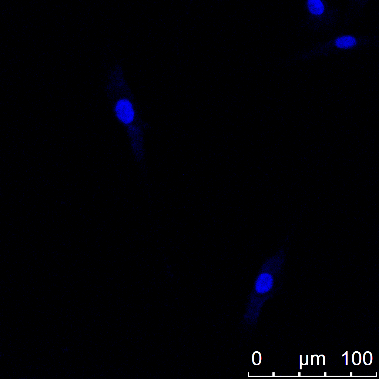

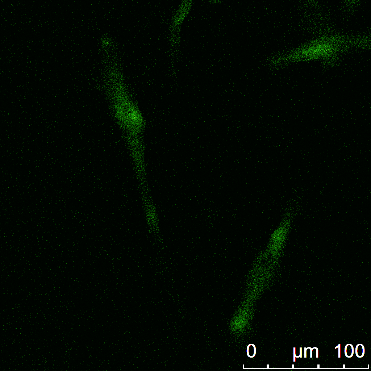

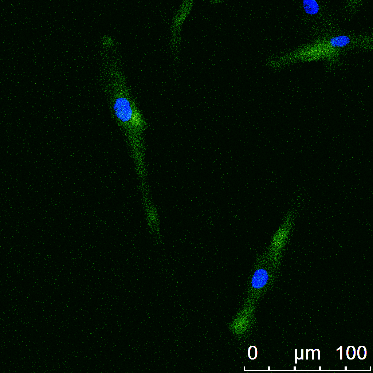


**HDMECs**

**DAPI PKH67 overlay**


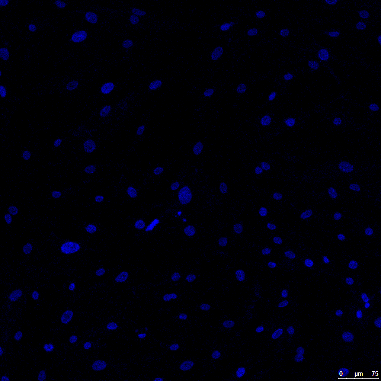

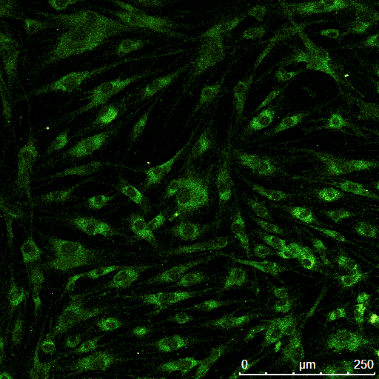

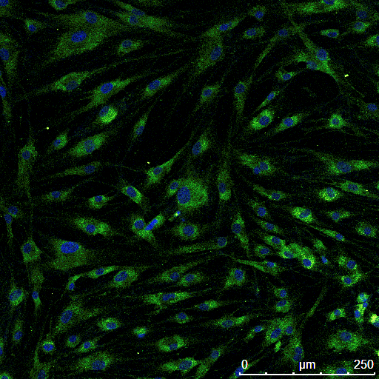

Supplement: Supplementary file 2 [file Table11.DOC]
