# Supplementary material for: Potential predictive and therapeutic applications of small extracellular vesicles-derived circPARD3B in osteoarthritis
Source: Front Pharmacol. 2022 Oct 19;13:968776. doi: 10.3389/fphar.2022.968776 (PMC9627215; doi:10.3389/fphar.2022.968776)
Supplement: Supplementary file 4 [file Table1.docx]

**Supplementary Figure 1**


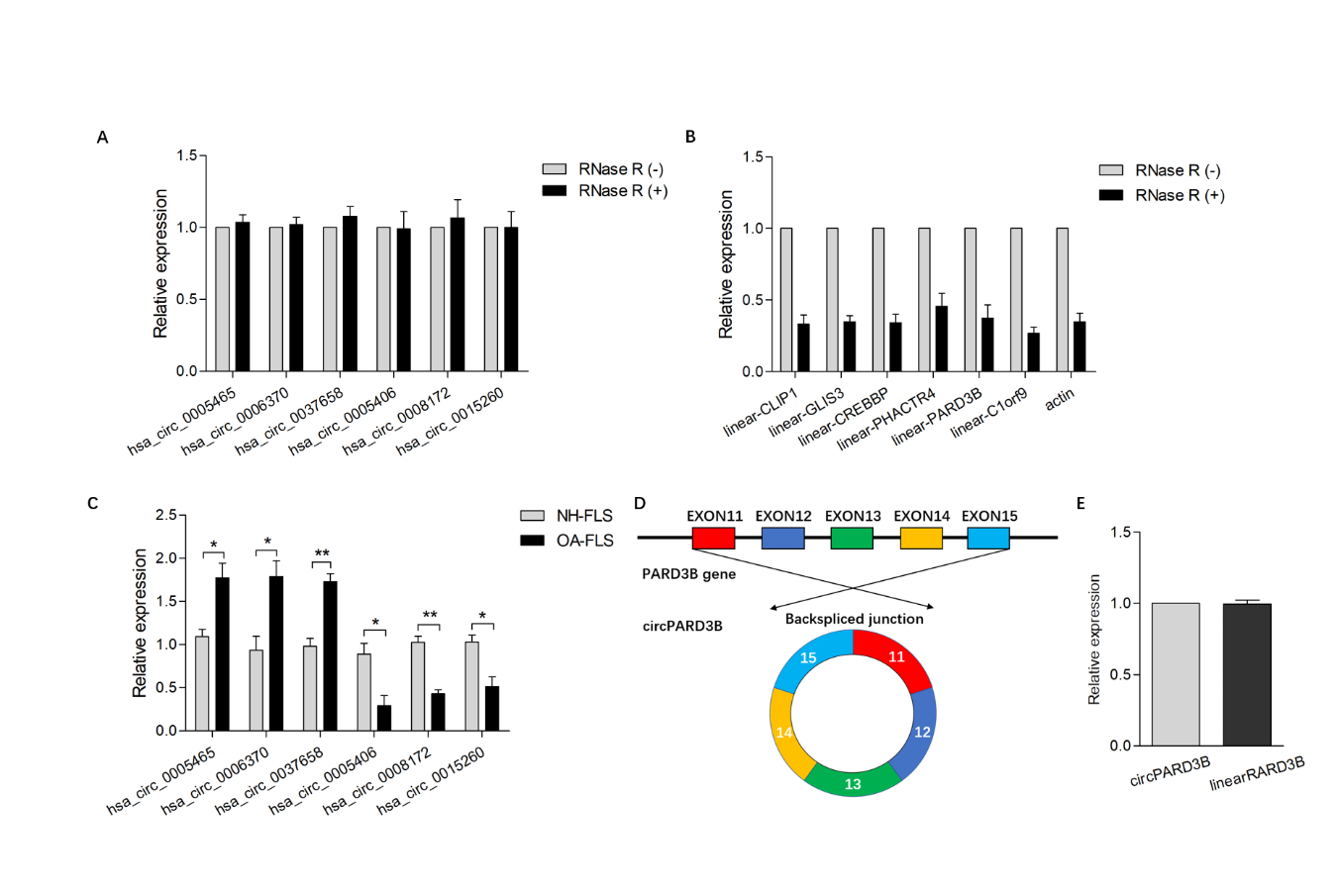


**Supplementary Figure 1. Confirmation of significantly up- or down-regulated circRNAs expressed in OA-FLS relative to NH-FLS.**

Linear and circular RNA expressed in FLS were determined by qPCR. According to the previous article (Xiang et al., 2019), we first selected 6 significantly up- or down-regulated circRNAs expressed in OA-FLS relative to normal human (NH-FLS), which were verified to resist RNase R’s digestion (A), while their linear forms were digested by RNase R (B). (C) Using RT-qPCR analysis of RNAs extracted from independent biological replicates of OA-FLS and NH-FLS from knee joint synovium, we found that 3 circRNAs (hsa_circ_0005465, hsa_circ_0006370 and hsa_circ_0037658) had significantly higher expressions, while 3 circRNAs (hsa_circ_0005406, hsa_circ_0008172 and hsa_circ_0015260) had significantly lower expressions. (D) Schematic illustration showing the circularization of PARD3B exon 11-15 to form circPARD3B. (E) No significant difference of expression level between linear PARD3B and circPARD3B were observed, and the “ratio” of linear vs circular RNA level were respectively 0.98, 0.96 and 1.05. n = 3 biologically independent samples in each group were determined. Data are expressed as the mean ± S.E.M. **P* < 0.05, ***P* < 0.01.

**Supplementary Figure 2**


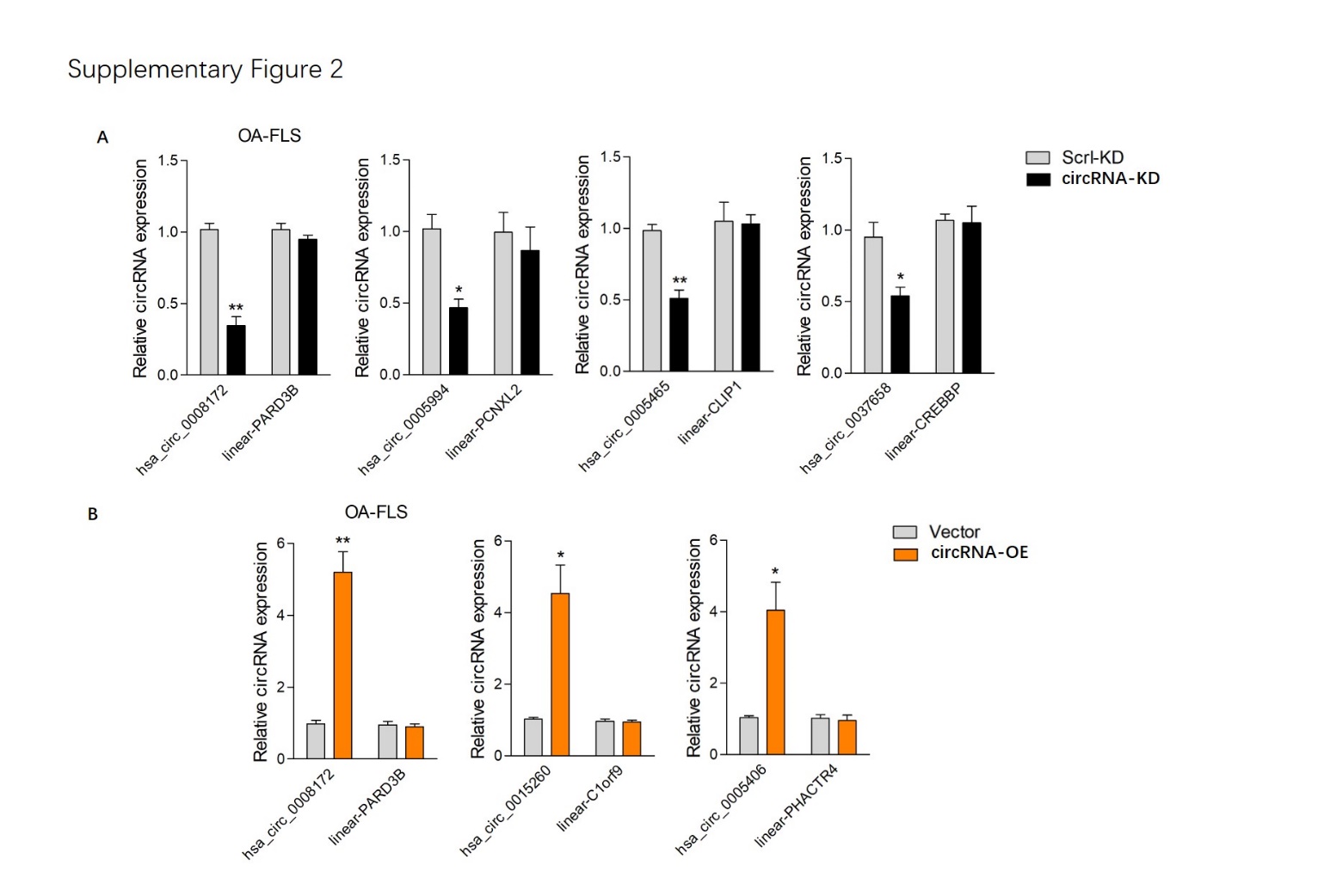

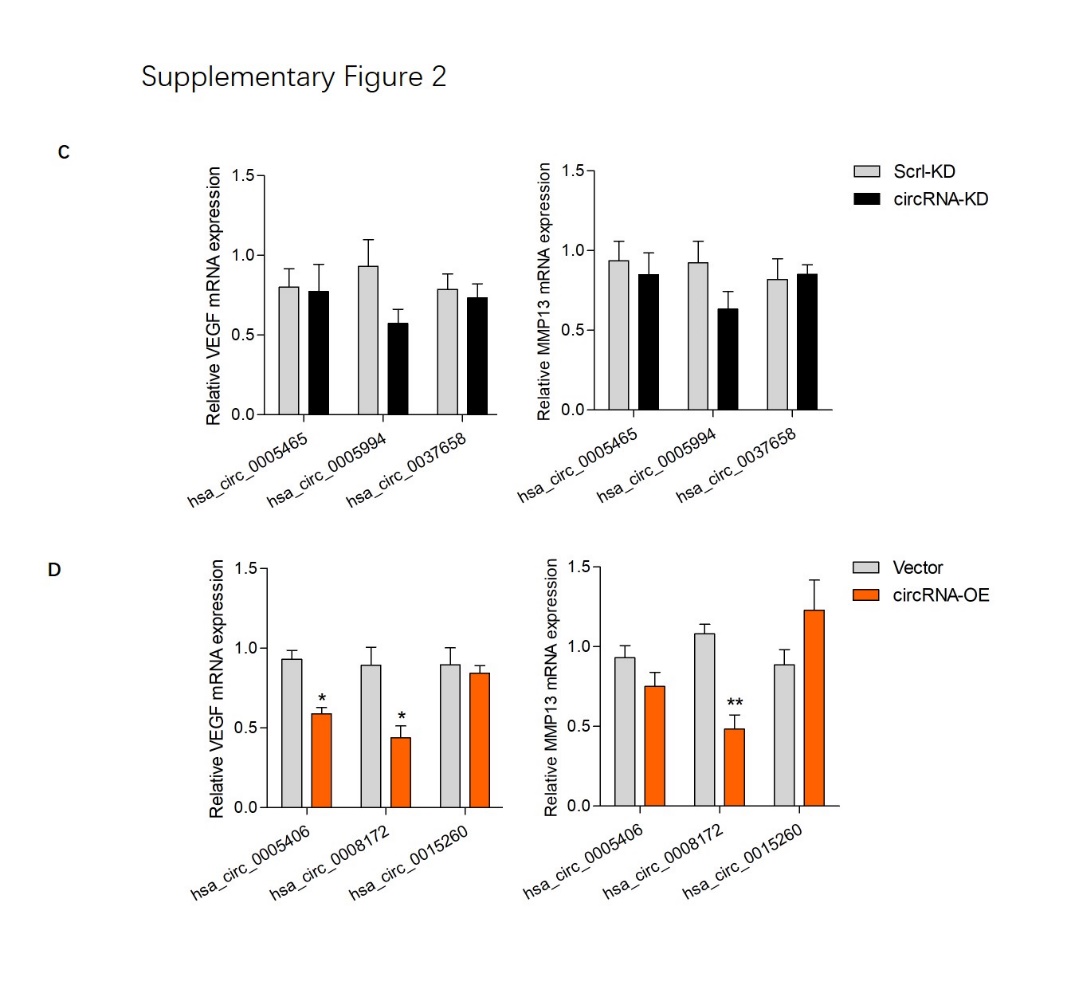


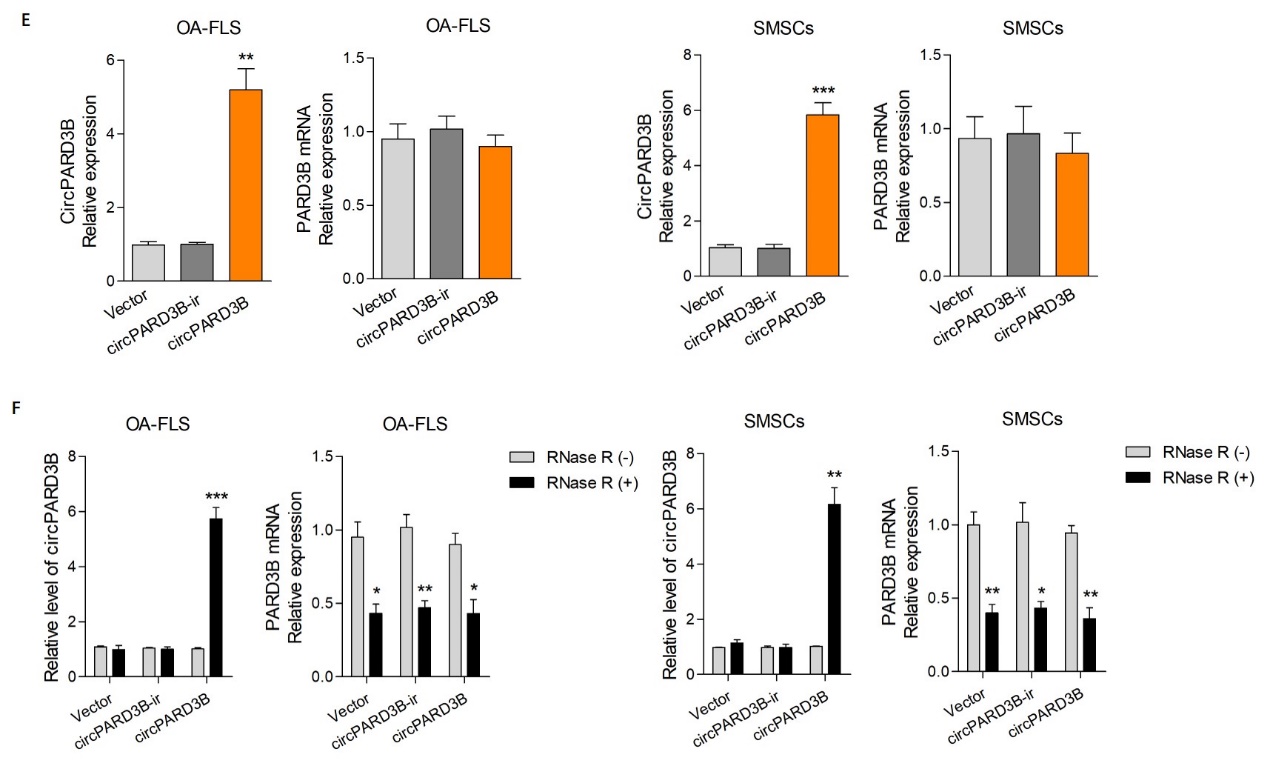


**Supplementary Figure 2. Overexpression or knockdown of differentially expressed circRNAs.**

The data shown in this figure were determined via qPCR analysis. Knockdown of hsa_circ_0008172, hsa_circ_0005994, hsa_circ_0005465, hsa_circ_0037658 (A), and Overexpression of hsa_circ_0008172, hsa_circ_0015260, hsa_circ_0005406 (B) were successfully achieved, while the expression of their linear forms were not significantly influenced. VEGF and MMP13 mRNA expression after circRNAs knockdown (C) or overexpression (D) were demonstrated. (E) Overexpression of human circPARD3B in OA-FLS and SMSCs respectively. (E) Overexpression efficiency of circPARD3B in OA-FLS (n=3) and SMSCs (n=3) by qPCR. The expression of PARD3B mRNA exihibited no significant alterations in response to circPARD3B overexpression in OA-FLS (n=3) and SMSCs (n=3) transfectants. (F) CircPARD3B that was overexpressed from the Adenovirus vectors were verified to resist RNase R’s digestion, while the linear forms were digested by RNase R. Data are expressed as the mean ± S.E.M. n = 3, each group; **P* < 0.05, ***P* < 0.01.

**Supplementary Figure 3**


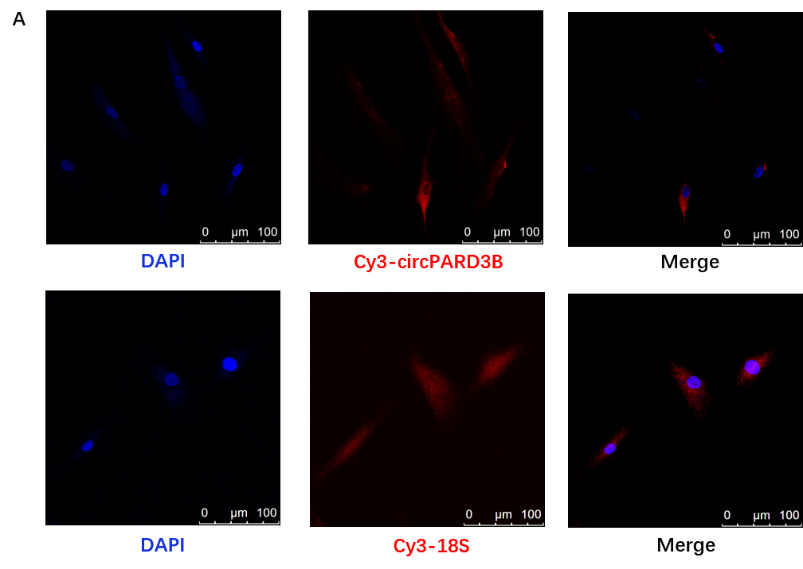


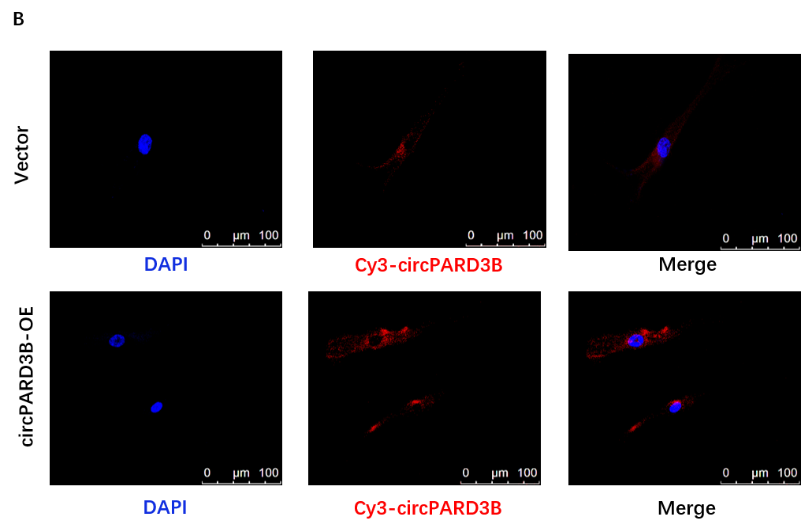


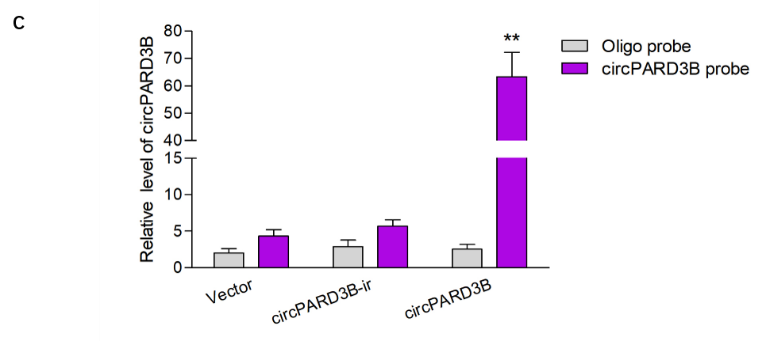


**Supplementary Figure 3. Location of circPARD3B in cytoplasm.**

(A) Fluorescence in situ hybridization (FISH) results showed that circPARD3B transcript signals were mostly located in the cytoplasm of OA-FLS. (B) CircPARD3B transcript signals in OA-FLS which overexpressed circPARD3B were mostly located in the cytoplasm of OA-FLS. (C) Lysates prepared from OA-FLS transfected with vector, circPARD3B-ir or circPARD3B were subjected to RNA pull-down assays with a circPARD3B probe or an oligo control probe. The captured pellets were examined by qPCR. The relative level of circPARD3B was normalized to the input (n=3).

**Supplementary Figure 4**


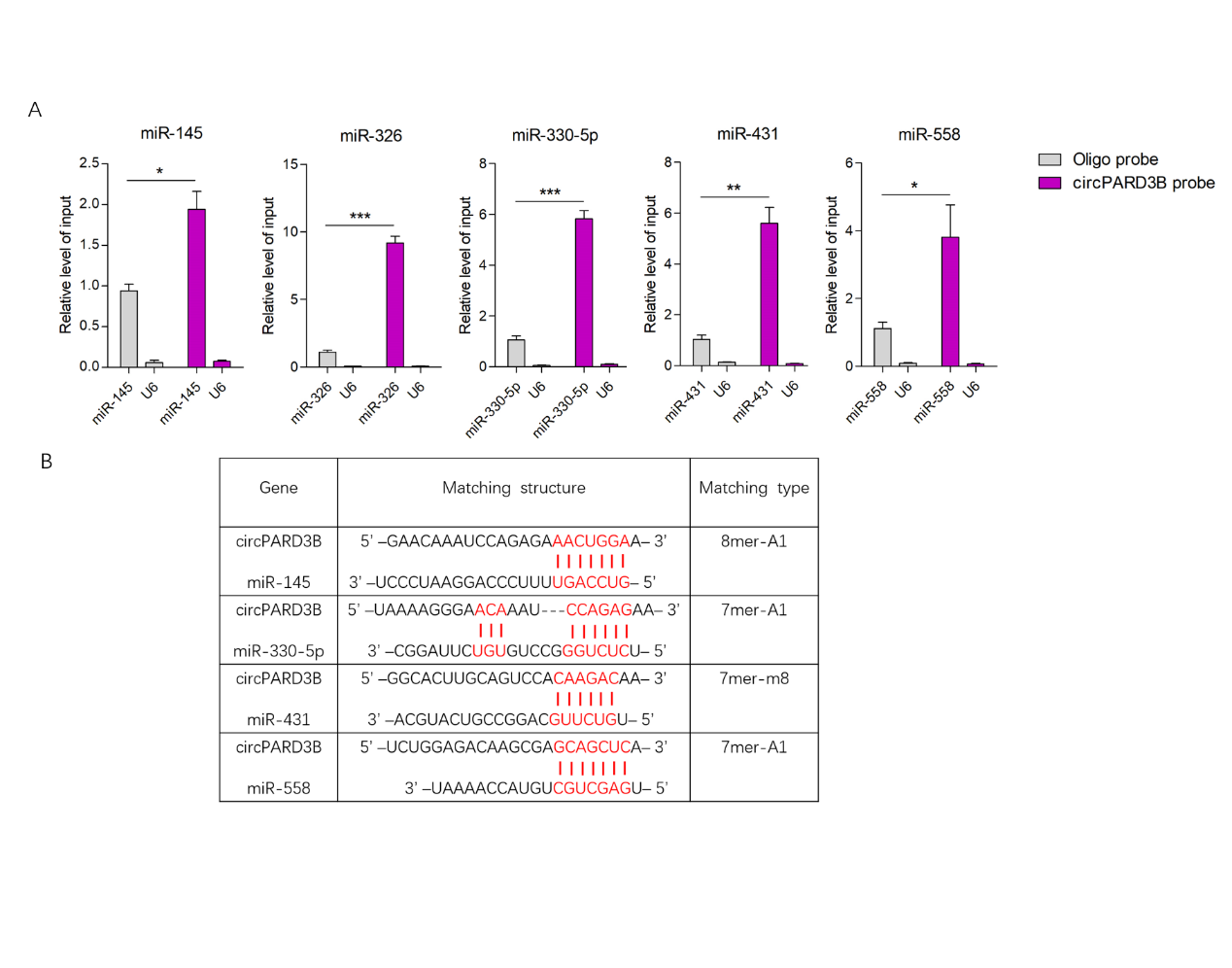


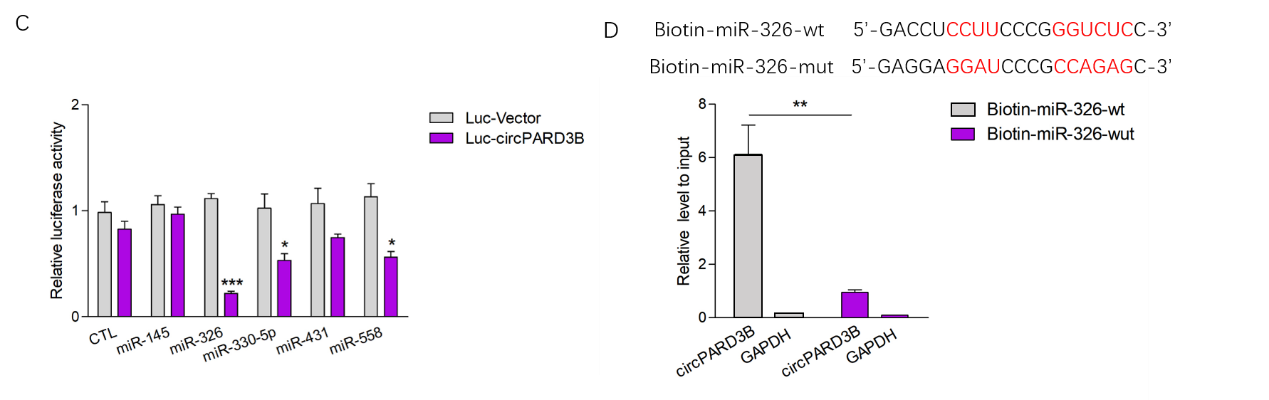


**Supplementary Figure 4. MiRNAs selection influenced by circPARD3B.**

(A) RT-qPCR quantification of the circPARD3B-bound 5 miRNA candidates pulled down in the cell lysates. U6 were used as internal controls of qPCRs. (B) At least one miRNA-binding site defined by the Arraystar Proprietary Algorithms was confirmed using the CircInteractome database. The schematic graph illustrated binding sites between circPARD3B and miR-145, miR-330-5p, miR-431, miR-558 predicted by bioinformatics methods. (C) Relative luciferase activity of circPARD3B luciferase reporter after co-transfection with indicated 5 miRNA mimics. (D)A biotinylated miR-326 probe or a probe that was mutated in the seed region to pull down circPARD3B were employed. Biotin-coupled miR-326 captured more circPARD3B than biotin-coupled mutant probe, verifying that miR-326 could bind to circPARD3B in OA-FLS. Data are expressed as the mean ± SEM. n = 3, each group; * *P* < 0.05, ** *P* < 0.01, *** *P* < 0.001.

**Supplementary Figure 5**


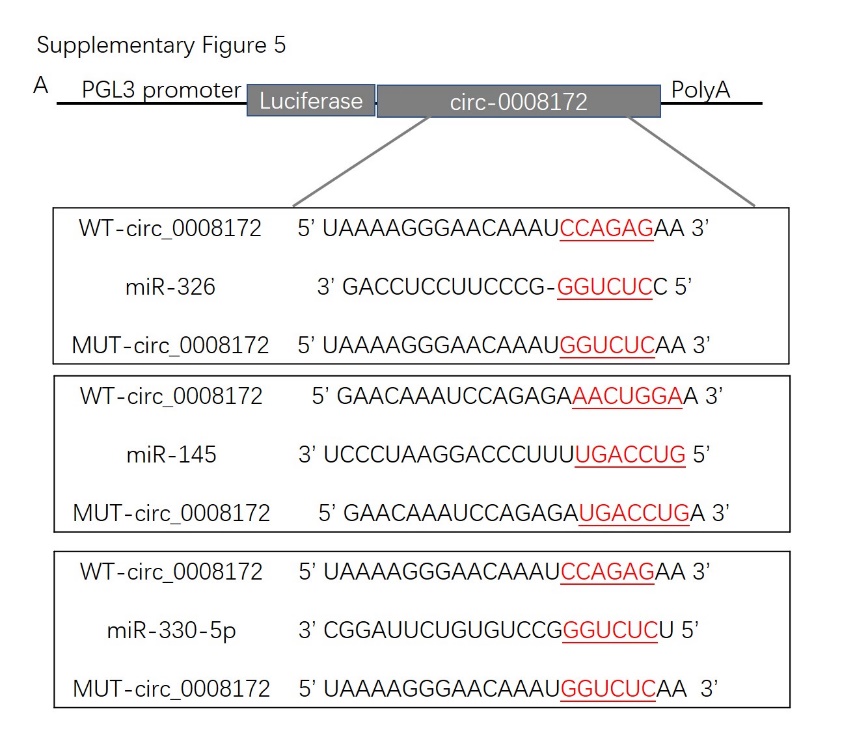


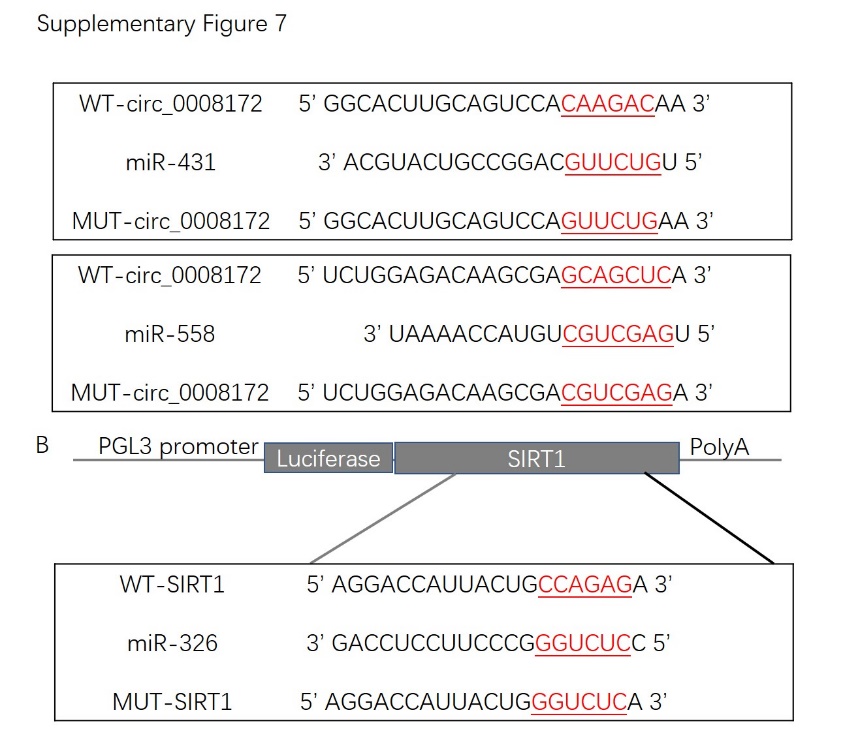


**Supplementary Figure 5. Wild type and mutated putative binding sites of circPARD3B and SIRT1 for luciferase reporter assay.**

**Supplementary Figure 6**


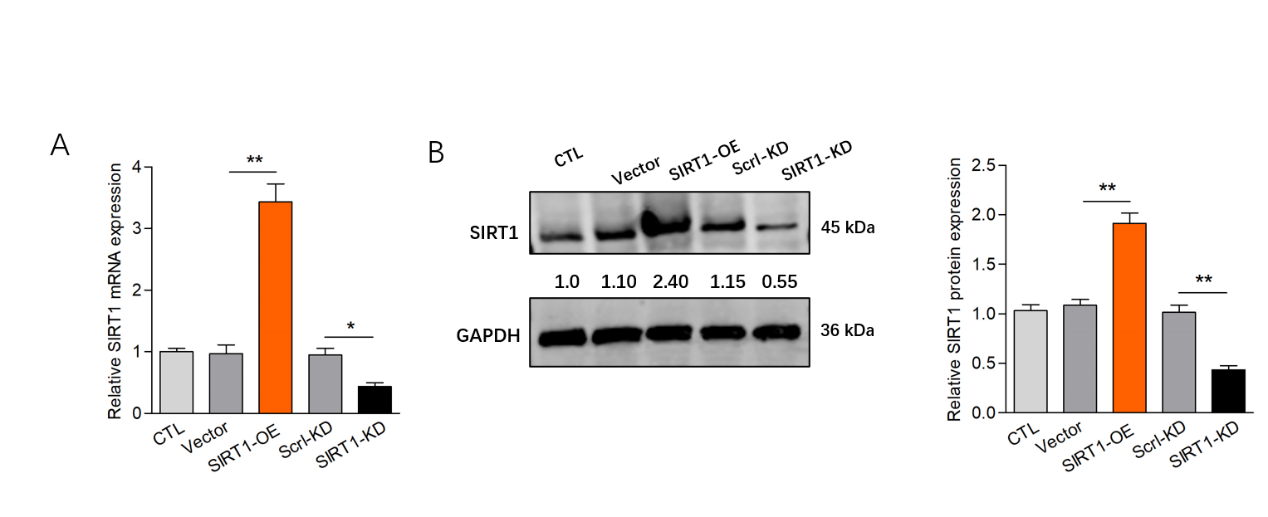


**Supplementary Figure 6. Overexpression and silencing of SIRT1.**

SIRT1 mRNA (A) and protein (B) expression were significantly upregulated by SIRT1 overexpression and downregulated by silencing. mRNA (A) and protein (B) expression were respectively determined by qPCR and western blot analysis. Data are expressed as the mean ± SEM. n=3 in each group; * *P* <0.05, ** *P* <0.01.

**Supplementary Figure 7**


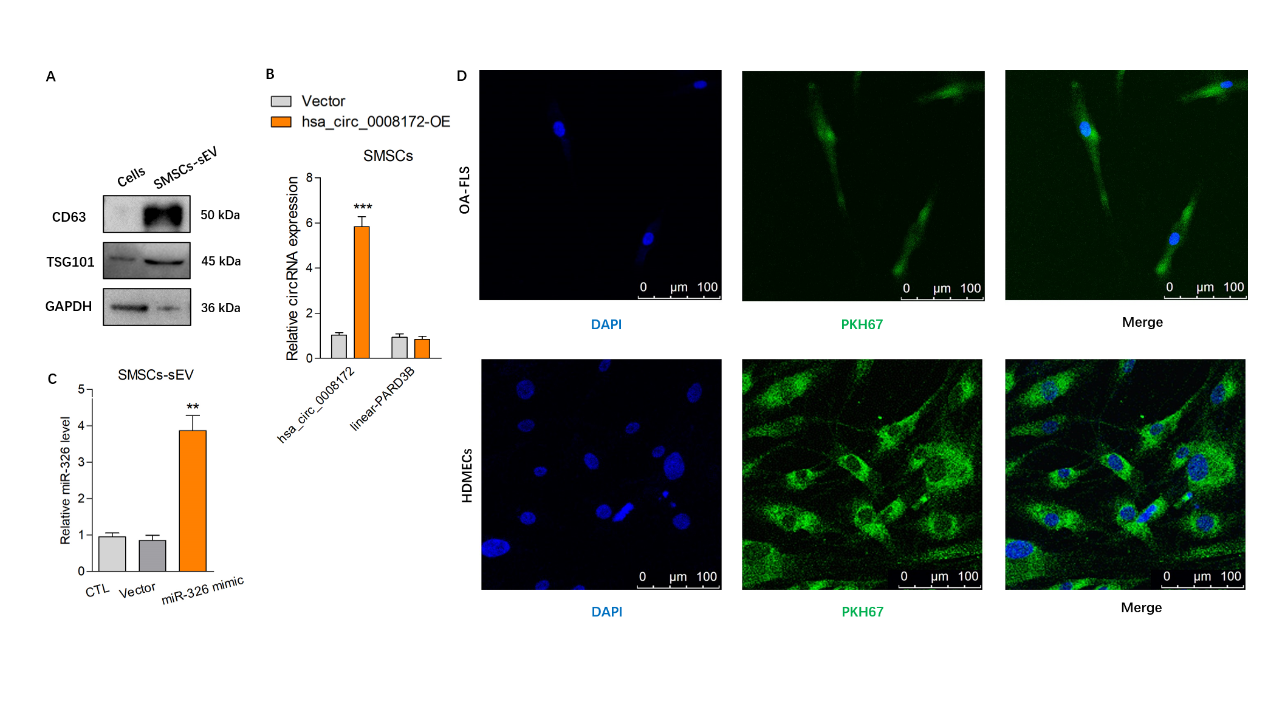


**Supplementary Figure 7.**

(A) Positive of conventional specific markers of CD63 & TSG101, and negative of GAPDH via WB analysis. (B) Overexpression of hsa_circ_0008172 (circPARD3B) was successfully achieved via adenovirus vector encoding circPARD3B (hsa_circ_0008172-OE) infected SMSCs, while the expression of their linear forms were not significantly influenced. (C) MiR-326 level in miR-326-mimic-SMSCs-sEV was confirmed by qPCR. (D) SMSCs-sEV were labeled with PKH67 (green) and co-cultured with OA-FLS or HDMECs for 6h. Fluorescence microscope analysis showed the stained sEV could be endocytosed into cells. Data are expressed as the mean ± SEM. n=3 in each group;*** *P* <0.001.

**Supplementary Figure 8**

**
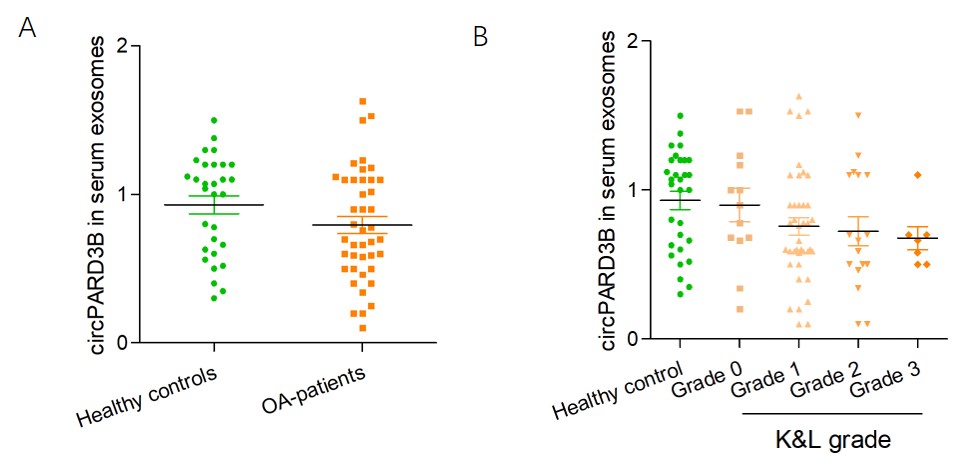
**

**Supplementary Figure 8. Serum sEV circPARD3B levels in OA patients and Non-OA controls.**

sEV circPARD3B levels were detected by qPCR. (A) There was no significant difference for serum sEV circPARD3B levels between OA patients and non-OA controls. (B) There was no significant difference for serum sEV circPARD3B levels in patients with different K&L grades. Data are expressed as the mean ± SEM. n=3 in each group.

**Supplementary Figure 9**


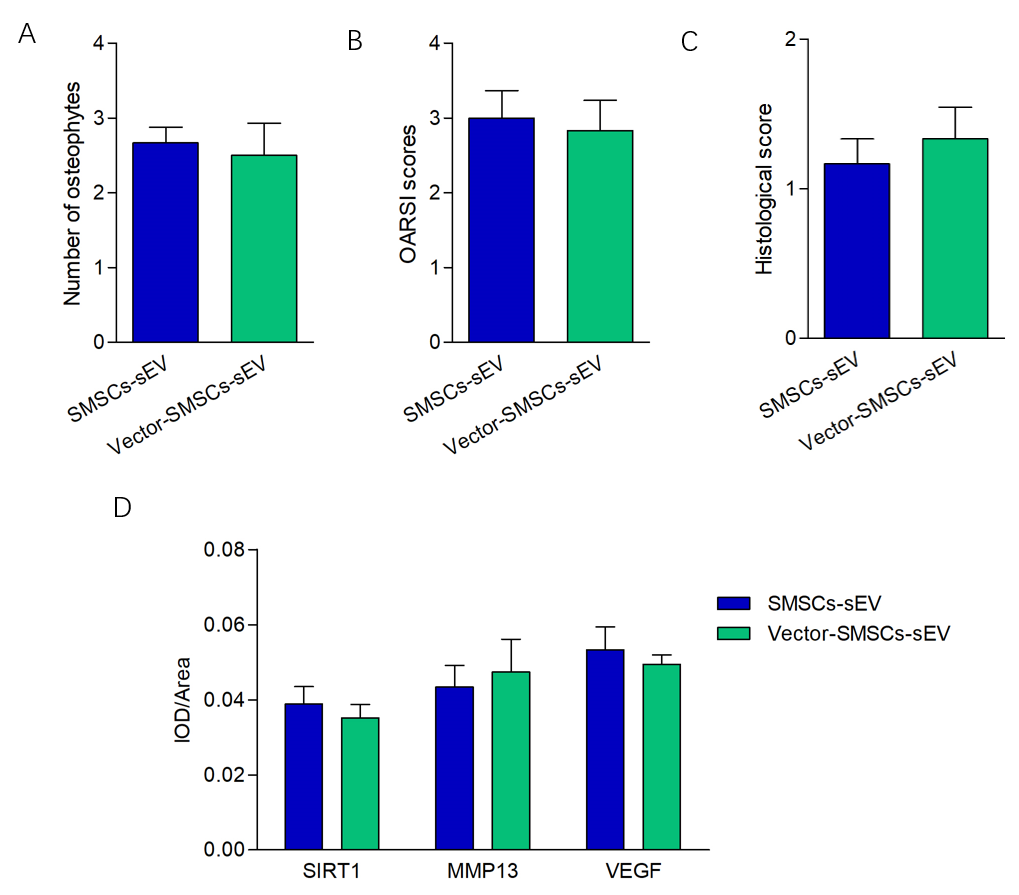


**Supplementary Figure 9. Effects of Vector-SMSCs-sEV treatment in animal model of collagenase‑induced osteoarthritis.**

No significant difference in osteophytes formation (A), OARSI scoring (B), H&E (C) and immunohistochemical staining (D) was observed between SMSCs-sEV and Vector-SMSCs-sEV treatment. Data are expressed as the mean ± SEM. n=6 in each group.

**Supplementary Table 1 (Table S 1). Primers and probes used in the study.**

| Gene | Sequence |
| --- | --- |
| hsa_circ_0005465 | 5’–GGCTGGCTCAATCAAGAAAGG–3′  3′–CTCGCTCCCCAACTCGAAAG–5’ |
| hsa_circ_0006370 | 5’–AACGCCCGCTATAAACTGCT– 3′  3′–CCATTCTGAGAGCCGTGGTT–5’ |
| hsa _circ_0037658 | 5’–GGAAGTGGTGGCTATGAGTCAG–3′  3′–TGTCAATTTGAAACTTAAAAAGCAG–5’ |
| hsa_circ_0005406 | 5’–GACCATGCCTGGCTCTGT–3′  3′–GACCATGCCTGGCTCTGT–5’ |
| hsa_circ_0008172 | 5’–TTCCAGATCAGCACATCAACT–3′  3′–TGAGCTGCTCGCTTGTCTC–5’ |
| hsa_circ_0015260 | 5’–TCCAGGGCGAGAATGTGAC–3′  3′–TCCAGGGCGAGAATGTGAC–5’ |
| miR-326 | 5’–-CATCTGTCTGTTGGGCTGGA–3′  3′–AGGAAGGGCCCAGAGGCG–5’ |
| miR-145 | 5’–CGGAATTCAAGGTCACTAGAGCCTGGGAAC–3′  3′–CGCGGATCCTTCAACCACTGTGTCTTGAGAC–5’ |
| miR-330-5p | 5’–TCTCTGGGCCTGTGTCTTAG–3′  3′–CAGTGCGTGTCGTGGAGT–5’ |
| miR-431 | 5’–TGTCTTGCAGGCCGTCATG–3′  3′–GCTGTCAACGATACGCTACCTA–5’ |
| miR-558 | 5’–TAGACTCTATTATAGTTTCCTG–3′  3′–GTTTGTGGTATTTTGGTA–5’ |
| U6 | 5′–GTAGATACTGCAGTACG–3′  3′–ATCGCATGACGTACCTGAGC-5′ |
| CLIP1 | 5’–AGAAGACGCTGCTGGACACAGA–3’  3’–TGGCATCTTCCGCTGTTTGAGC–5’ |
| GLIS3 | 5’–GTTCAGCGACTGGGACTCATT–3’  3’–CCCTCTGTAAGCTAGGACTGAT–5’ |
| CREBBP | 5’–CGGCTCTAGTATCAACCCAGG–3’  3’–TTTTGTGCTTGCGGATTCAGT–5’ |
| PHACTR4 | 5’–GAAGCAGACCAGCCCACTAC–3’  3’–CTTGCCAAAGCCTGAGAACTT–5’ |
| PARD3B | 5’–CAACTGGCCGCATTTAAGCC–3’  3’–CAGTGGAGTGCCTAGTTTTAGAG–5’ |
| C1orf9 | 5’–GACACTCCCTACAGTTGATTTGC–3’  3’–GGTGGACTGACAAAGGAAGGT–5’ |
| SIRT1 | 5’–GTCACACTTACGACAGAGCAGC–3’  3’–TTTCTCCAGTACATACACAAC–5’ |
| VEGF | 5’–TTCTGGGCTGTTCTCGCTTC–3’  3’–CTCTCCTCTTCCTTCTCTTCTTCC–5’ |
| MMP13 | 5’–TGACTGGCAAACTTGAGACGATA–3’  3’–AGGGTGTAATCACCATCTGTAG–5’ |
| GAPDH | 5’–GCACCGTCAAGGCTGAGAAC–3′  3′–TGGTGAAGACGCCAGTGGA–5’ |

Biotin-coupled probe pull down assay

| circPARD3B probe | 5′–AAGAGCATGGACCTTGAAAGGAGAACCTGA–3′ |
| --- | --- |
| Oligo probe | 5′–TGTCTGCAATATCCZGGGTTTCCGATGGCA–3′ |
| miR-326-wt probe | 5’-GACCUCCUUCCCGGGUCUCC-3’ |
| miR-326-mut probe | 5’- GAGGAGGAUCCCGCCAGAGC -3’ |

FISH probes

| hsa_circ_0008172 | 5′–CY3–TCGTACCTGGAACTTTCCTCTTGG–3′ |
| --- | --- |

siRNAs

| Gene | Sequence |
| --- | --- |
| hsa_circ_0005465 | 5’–GACAGAGTATTGATTTAATCC–3’ |
| hsa_circ_0006370 | 5’–CAAGTGTACGGTCCCTTATTT–3’ |
| hsa_circ_0037658 | 5’–CGAACCGGACAGGTGCCTGGT–3’ |
| hsa_circ_0008172 | 5’–GCATGGACCTTGAAAGGAGAA–3’ |
| si-NC | 5’–UUCUCCGAACGUGUCACGU–3’ |
| CLIP1 | SS：GGAAGAAUCAAGACCUCAAGA  AS：UUGAGGUCUUGAUUCUUCCUU |
| GLIS3 | SS：CCUCAAAGACUGUGUUCAAAG  AS：UUGAACACAGUCUUUGAGGUG |
| CREBBP | SS：GAUGCUGCUUCCAAACAUAAA  AS：UAUGUUUGGAAGCAGCAUCUG |
| PARD3B | SS：GGUAUUAGAUGGAAGUGAAUG  AS：UUCACUUCCAUCUAAUACCUG |
| SIRT1 | SS：GAAGUUGACCUCAUUGU  AS：ACA AUGAGGUCA ACUUC |

| miR-326 mimic | RiboBio |
| --- | --- |
| miR-326 inhibitor | RiboBio |

**Supplementary Table 2 (Table S 2). Basic characteristics of OA patients and controls.**

| Characteristics | Controls  (Grade 0) (n=30) | KOA (Grade 0) (n=13) | KOA  (Grade 1)  (n=40) | KOA  (Grade 2)  (n=17) | KOA  (Grade 3)  (n=7) | *P* Value |
| --- | --- | --- | --- | --- | --- | --- |
| Age (years) | 59.3 (5.5) | 59.6 (7.0) | 60.3 (5.5) | 59.8 (5.7) | 61.0 (4.1) | 0.939 |
| Sex(male/female) | 7/23  (23.3%)^a^ | 3/13 (23.0%)^a^ | 8/32  (20%)^a^ | 3/14 (17.6%)^a^ | 2/5 (28.6%)^a^ | 0.971 |
| BMI (kg/m^2^) | 25.2 (3.5) | 23.9 (8.1) | 22.2 (9.7) | 24.3 (7.0) | 24.0 (3.9) | 0.563 |
| Symptom duration (years) | — | 9.8 (3.1) | 8.9 (2.9) | 9.1 (3.4) | 8.2 (2.2) | 0.688 |
| VAS (cm) | — | 5.5 (1.9) | 5.8 (1.8) | 5.9 (1.9) | 6.5 (1.1) | 0.629 |
| CRP (mg/dL) | 4.7 (3.6) | 8.2 (8.8) | 18.9(28.1) | 5.5 (4.9) | 19.1 (30.5) | 0.038 |
| ESR (mm/h) | 15.6 (9.1) | 35.8 (38.6) | 40.4(36.4) | 33.7 (35.9) | 38.0 (26.3) | 0.023 |

Data are mean (SD) or n (%).

^a^Males shown as a percentage.

KOA, knee osteoarthritis; BMI, body mass index; VAS, Visual Analogue Scale; CRP, C-reactive protein; ESR, erythrocyte sedimentation rate.

**Supplementary Table 3 (Table S 3). Correlations between serum sEV circPARD3B levels and clinical characteristics of KOA patients.**

| Characteristics | r值 | *P*值 |
| --- | --- | --- |
| Age (years) | -0.183 | 0.060 |
| BMI (kg/m^2^)* | 0.163 | 0.094 |
| Symptom duration (years) | 0.152 | 0.186 |
| VAS (cm) | 0.029 | 0.803 |
| CRP (mg/DL) | -0.104 | 0.288 |
| ESR (mm/h) | -0.260 | 0.007 |

KOA, knee osteoarthritis; BMI, body mass index; VAS, Visual Analogue Scale; CRP, C-reactive protein; ESR, erythrocyte sedimentation rate.

**Details on statistical methods:**

Figure 1. (A) Data were analyzed by unpaired two-tailed Student’s test. (B-G) One way ANOVA with Bonferroni post-hoc test was employed to determine statistical significance as indicated in the graphs.

Figure 2. (B) Data were analyzed by unpaired two-tailed Student’s test. (C) Statistical analyses were performed using two-way ANOVA followed by Bonferroni post-hoc test. (D, F) One way ANOVA with Bonferroni post-hoc test was employed to determine statistical significance as indicated in the graphs. (G) Statistical analyses were performed using two-way ANOVA followed by Bonferroni post-hoc test. (H) Data were analyzed by unpaired two-tailed Student’s test.

Figure 3. One way ANOVA with Bonferroni post-hoc test was employed to determine statistical significance as indicated in the graphs.

Figure 4. (D-I) Statistical analyses were performed using one-way ANOVA followed by Bonferroni post-hoc test.

Figure 5. (D) One way ANOVA with Bonferroni post-hoc test was employed to determine statistical significance as indicated in the graphs.

Figure 6. (E-H) Statistical analyses were performed using one-way ANOVA followed by Bonferroni post-hoc test.

Supplementary Figure 1. (A-C) Data were analyzed by unpaired two-tailed Student’s test. Correct for multiple comparisons using the Holm-Sidak method.

Supplementary Figure 2. (A-D, F) Statistical analyses were performed using two-way ANOVA followed by Bonferroni post-hoc test. (E) One way ANOVA with Bonferroni post-hoc test was employed to determine statistical significance.

Supplementary Figure 3. (C) Data were analyzed by one-way ANOVA followed by Bonferroni post-hoc test.

Supplementary Figure 4. (A-D, E) Statistical analyses were performed using two-way ANOVA followed by Bonferroni post-hoc test.

Supplementary Figure 6. (A-B) Data were analyzed by one-way ANOVA followed by Bonferroni post-hoc test.

Supplementary Figure 7. (A) Data were analyzed by two-way ANOVA followed by Bonferroni post-hoc test. (B) Data were analyzed by one-way ANOVA followed by Bonferroni post-hoc test.

Supplementary Figure 8. (A-B) One way ANOVA with Bonferroni post-hoc test was employed to determine statistical significance as indicated in the graphs.

Supplementary Figure 9. (A-D) Data were analyzed by unpaired two-tailed Student’s test.
