# Supplementary material for: Potential predictive and therapeutic applications of small extracellular vesicles-derived circPARD3B in osteoarthritis
Source: Front Pharmacol. 2022 Oct 19;13:968776. doi: 10.3389/fphar.2022.968776 (PMC9627215; doi:10.3389/fphar.2022.968776)

**Figure 1D.**

***Demonstrated pictures in Figure.***


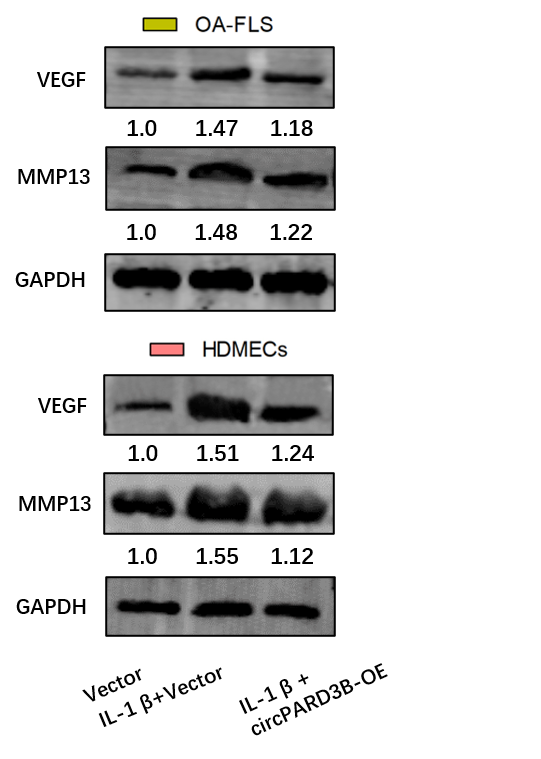


***Figure legends.***The effects of circPARD3B overexpression on VEGF/MMP13 protein expression in co-cultured OA-FLS or HDMECs were significantly upregulated after IL-1β induction, and then significantly downregulated by circPARD3B overexpression.

***Original images***

**OA-FLS-VEGF**


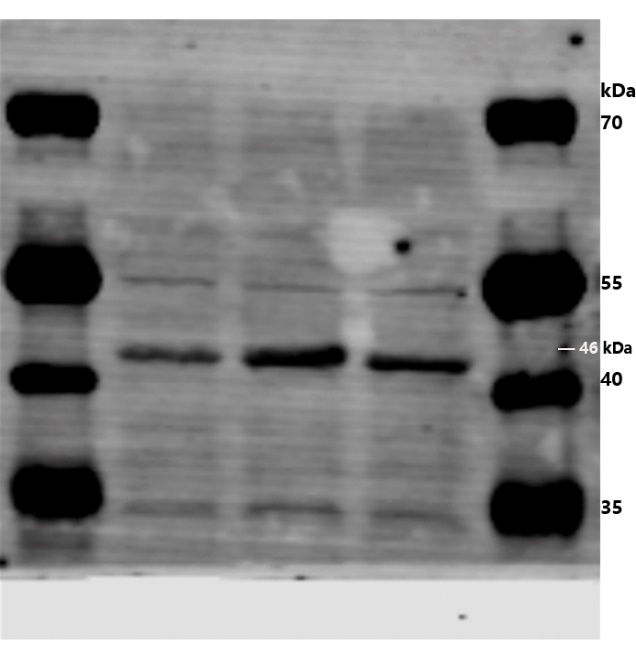


**OA-FLS-MMP13**


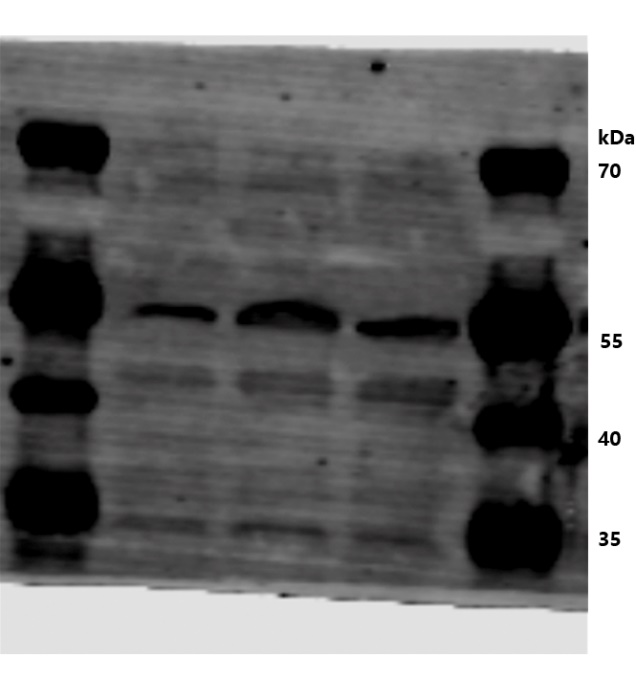


**OA-FLS-GAPDH**


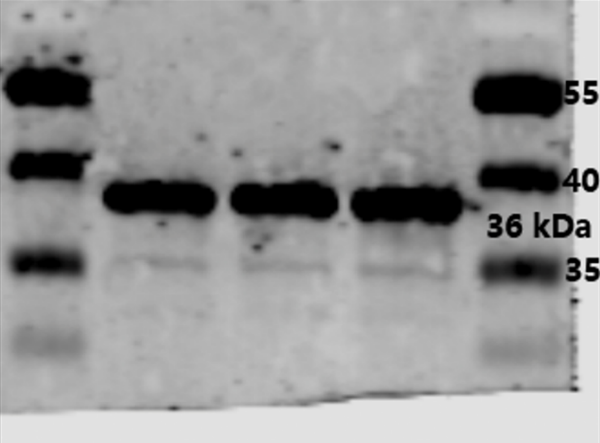


**HDMECs-VEGF**


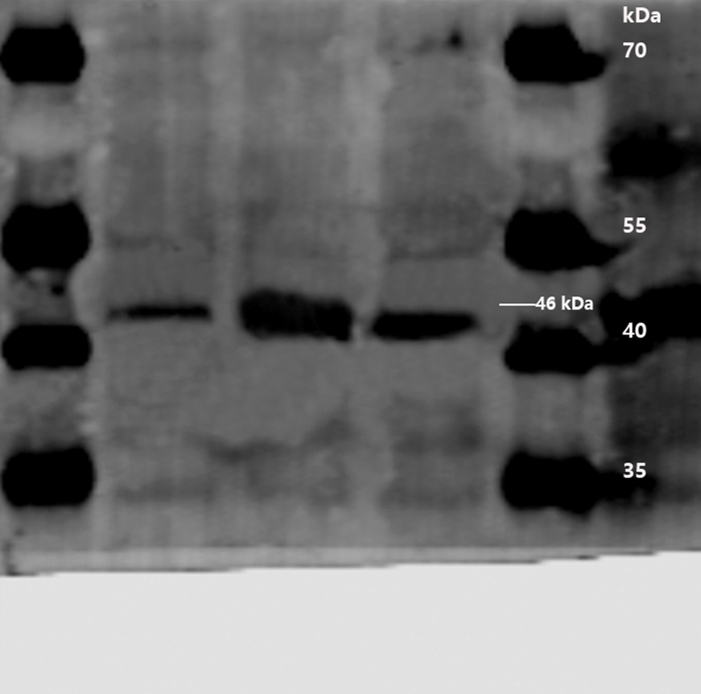


**HDMECs-MMP13**


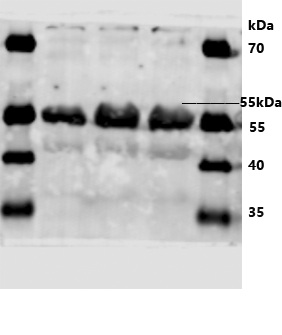


**HDMECs-GAPDH**


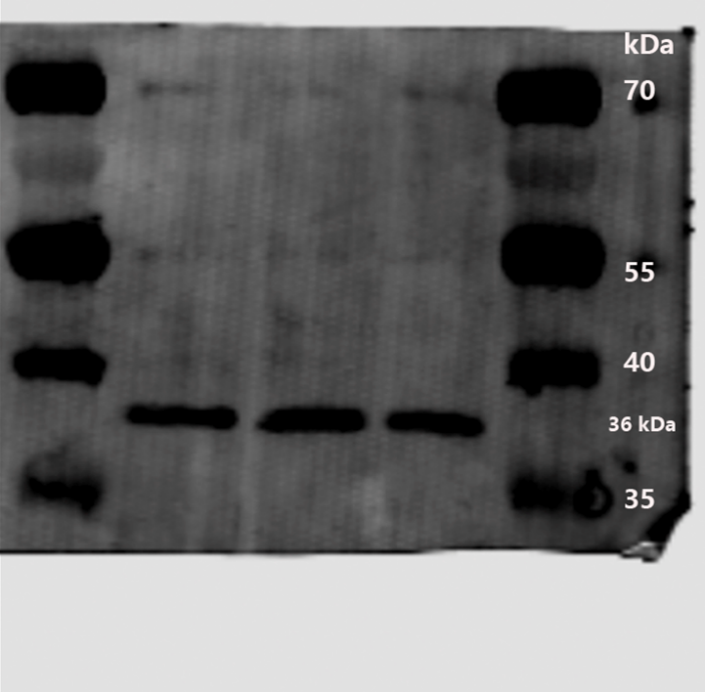


**Figure 3B.**

***Demonstrated pictures in Figure.***


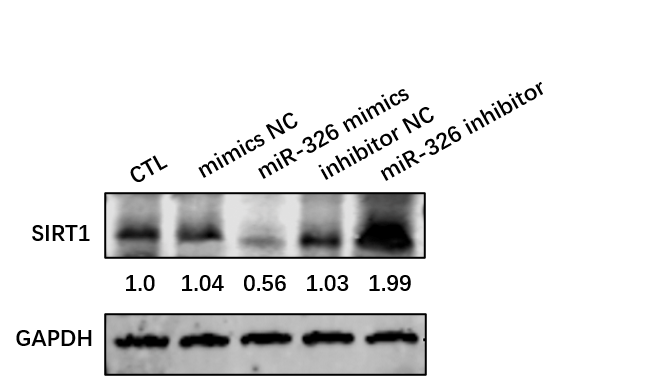


***Figure legends.***

SIRT1 protein expression were significantly down-/up-regulated by miR-326 mimics/inhibitor.

***Original images***

**SIRT1**


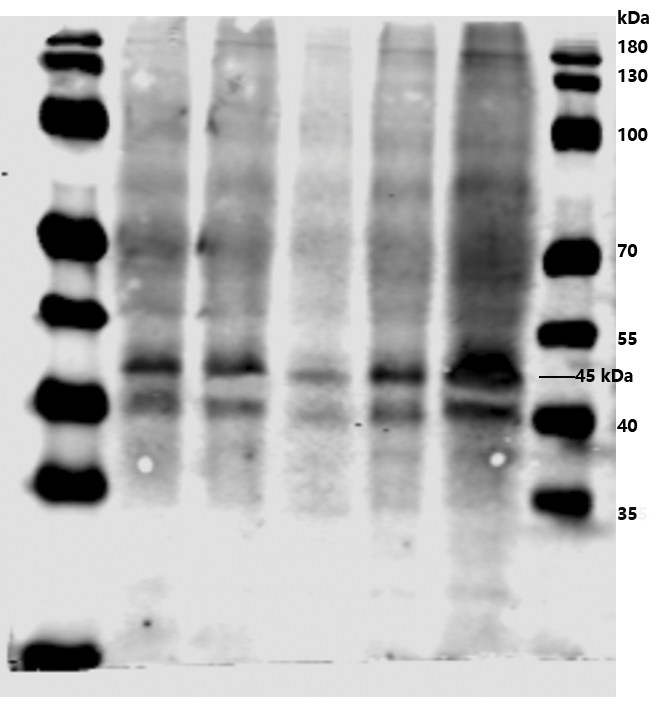


**GAPDH**


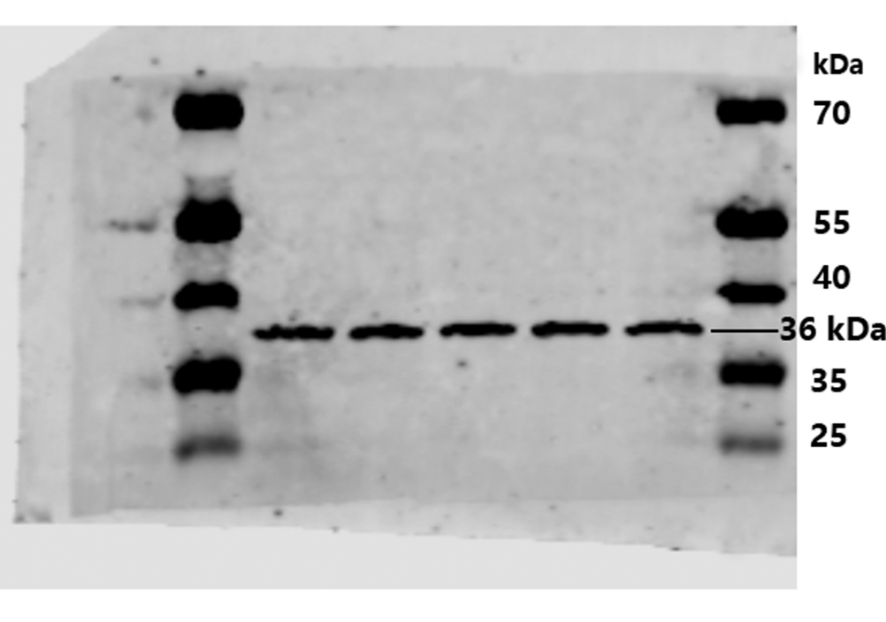


**Figure 3D.**

***Demonstrated pictures in Figure.***


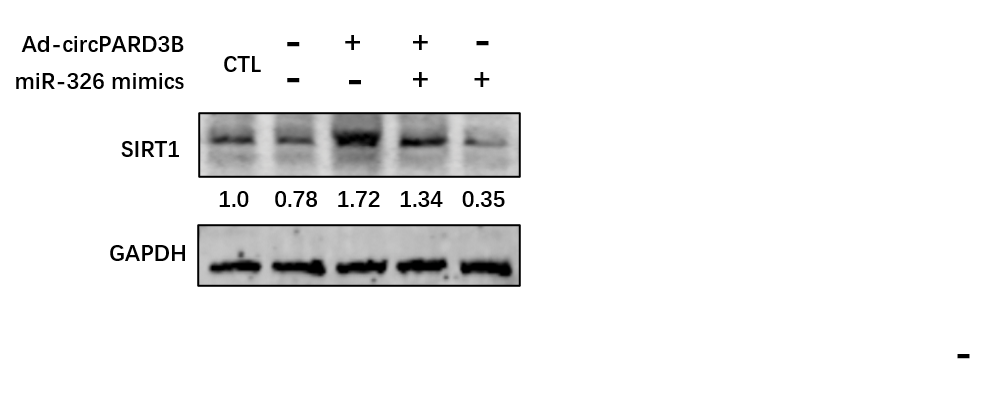


***Figure legends.***

Co-effects of circPARD3B and miR-326 on SIRT1 expression in OA-FLS were evaluated by detection of SIRT1 protein expression. SIRT1 expression were significantly elevated/reduced by circPARD3B overexpression/miR-326 mimics compared with those of each control group. Moreover, the significant downregulation of SIRT1 expression repressed by miR-326 mimics could be obviously rescued by circPARD3B overexpression, in other words, the significant upregulation of SIRT1 expression induced by circPARD3B overexpression could be significantly inhibited by miR-326 mimics.

***Original images***

**SIRT1**


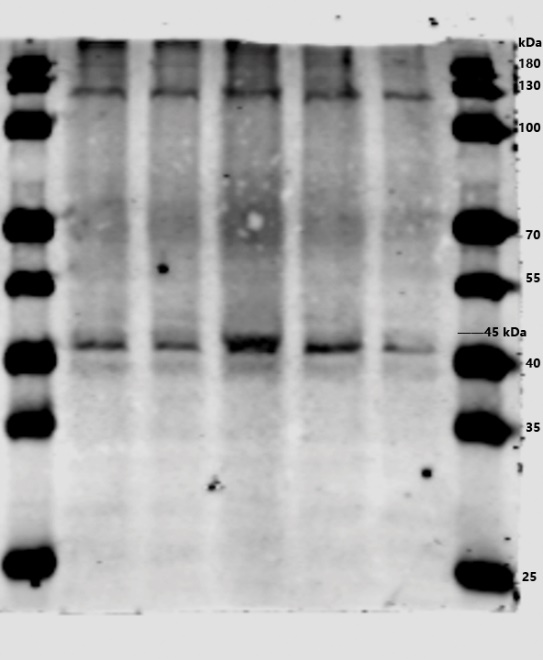


**GAPDH**


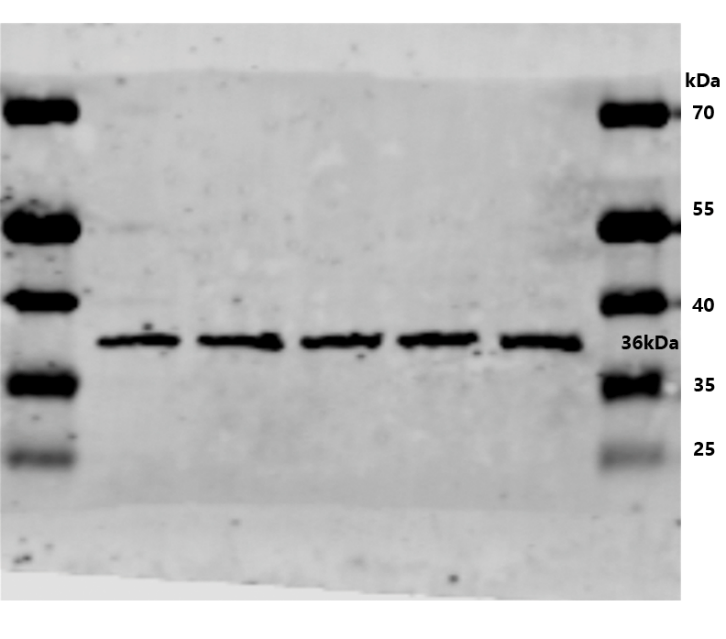


**Figure 3F.**

***Demonstrated pictures in Figure.***


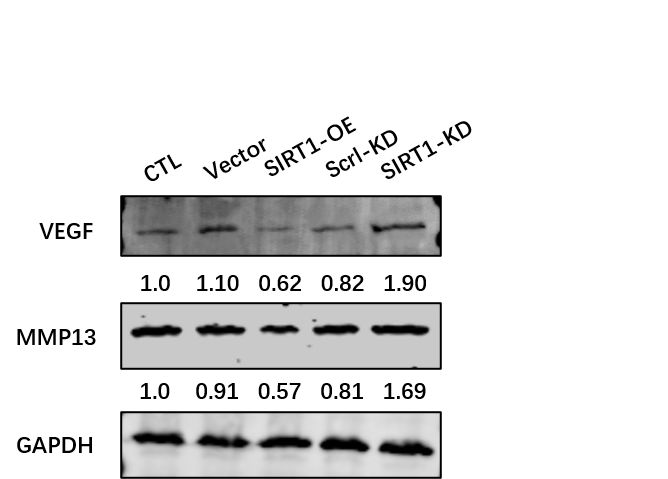


***Figure legends.***

The protein (F) expression of VEGF/MMP13 in OA-FLS were significantly reduced/elevated by SIRT1 overexpression/knockdown.

***Original images***

**VEGF**


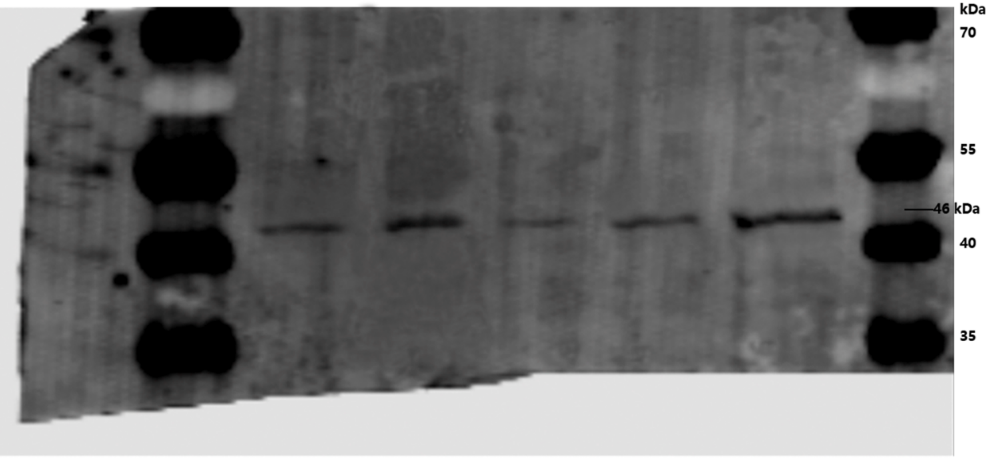


**MMP13**


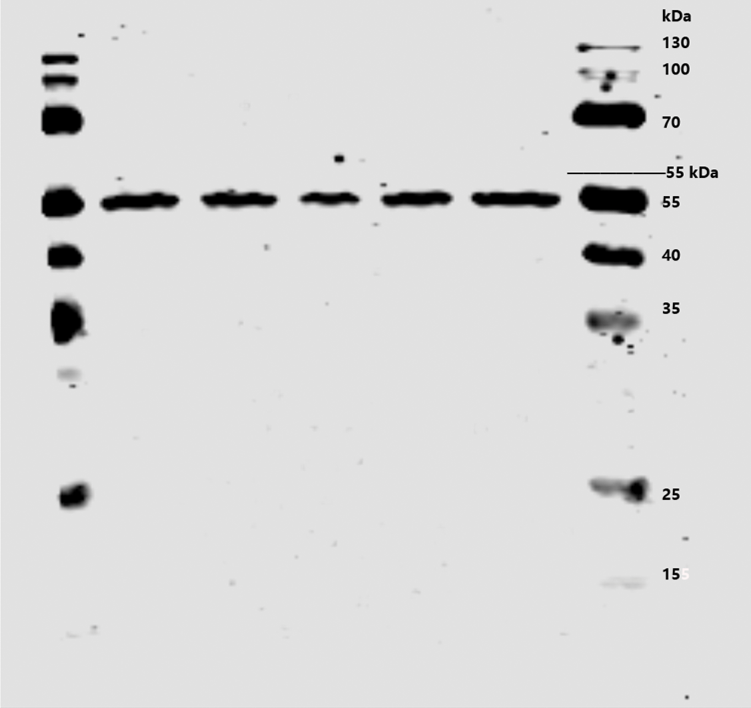


**GAPDH**


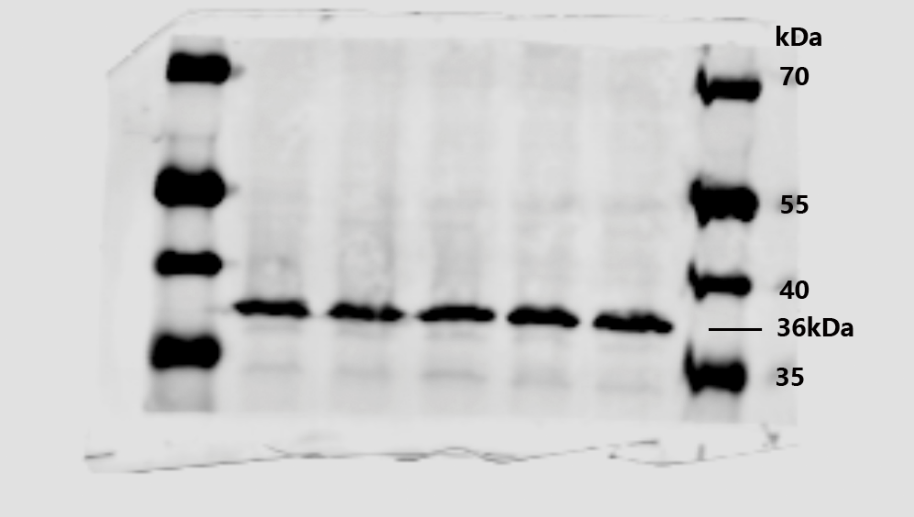


**Figure 3H.**

***Demonstrated pictures in Figure.***


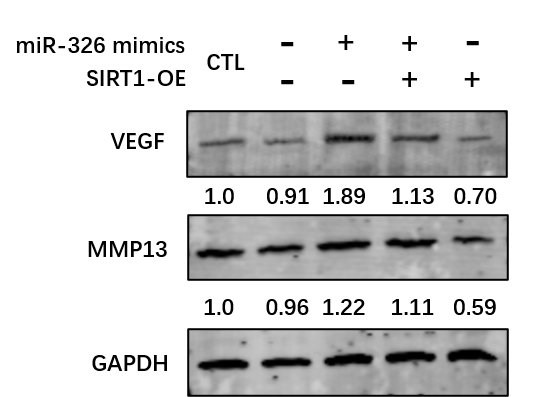


***Figure legends.***

MiR-326 mimics significanly upregulated VEGF/MMP13 protein expression, and these increasement could be signicantly suppressed by SIRT1 overexpression.

***Original images***

VEGF


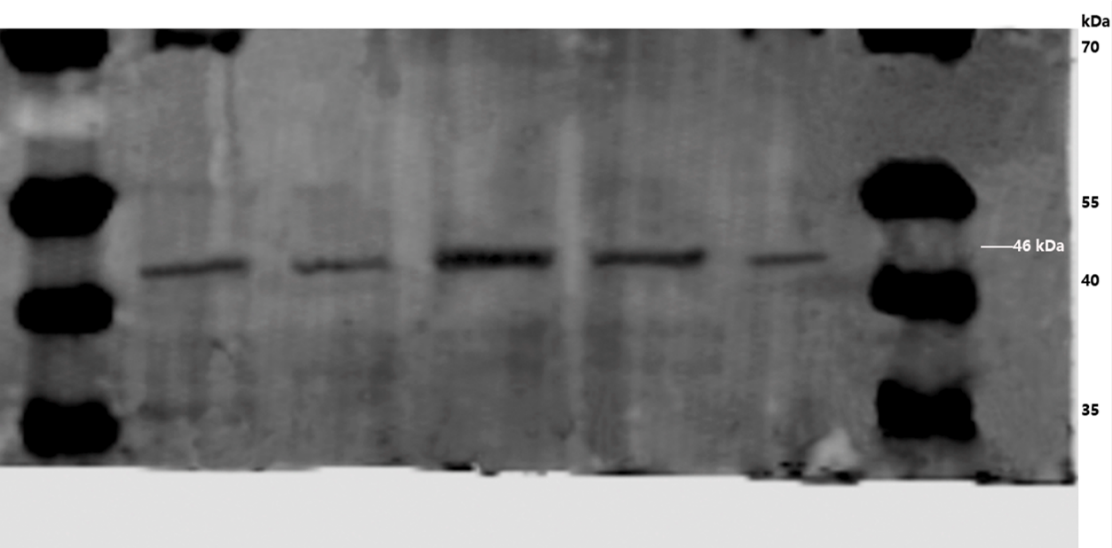


MMP13


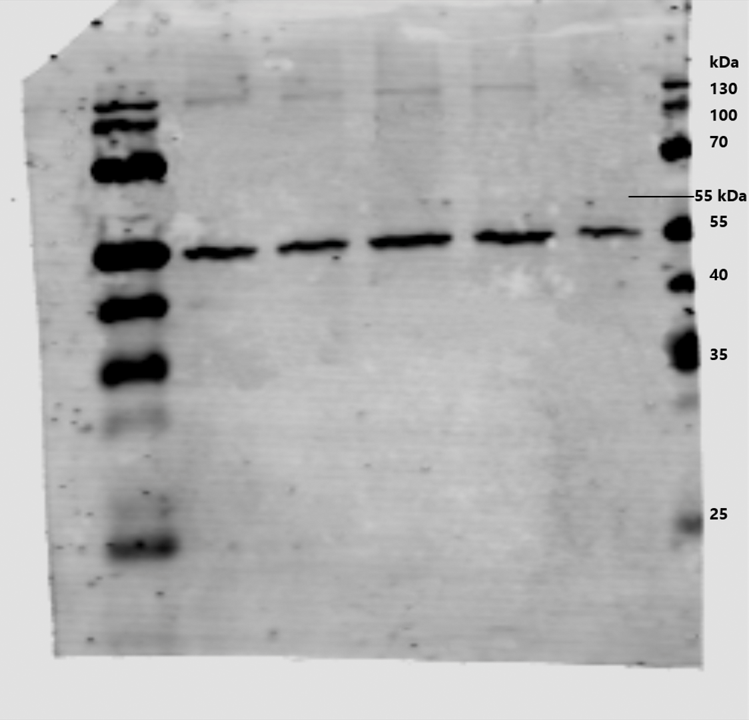


GAPDH


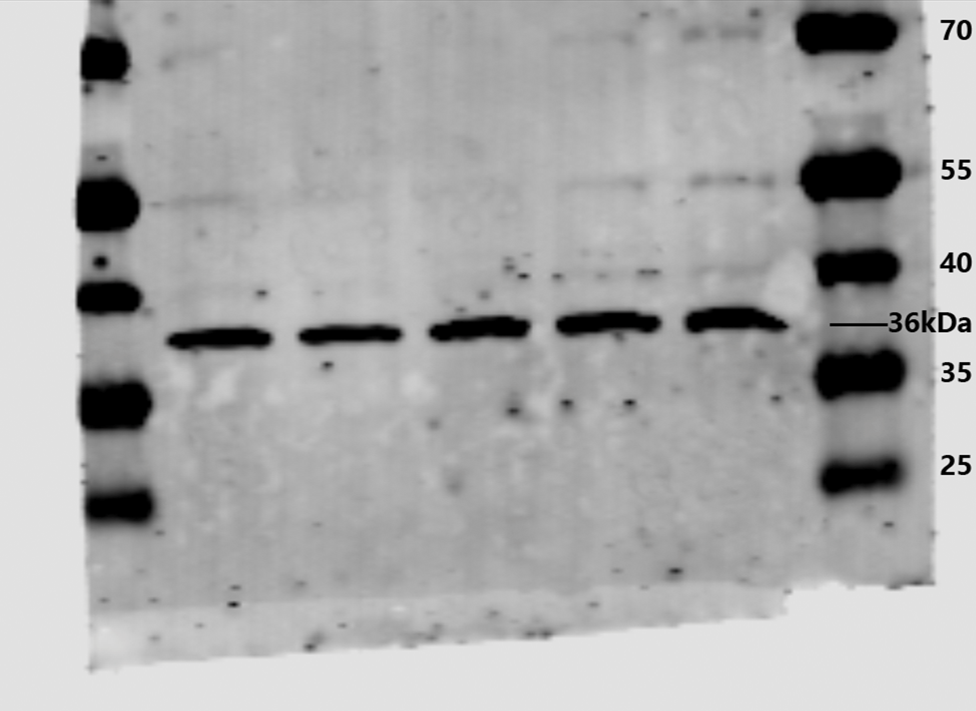


**Figure 4C.**

***Demonstrated pictures in Figure.***


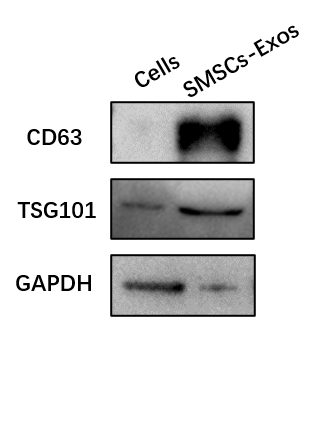


***Figure legends.***

Positive of conventional specific markers of CD63 and TSG101, and negative of GAPDH via western blot analysis.

***Original images***

**CD63 (Left two lane)**


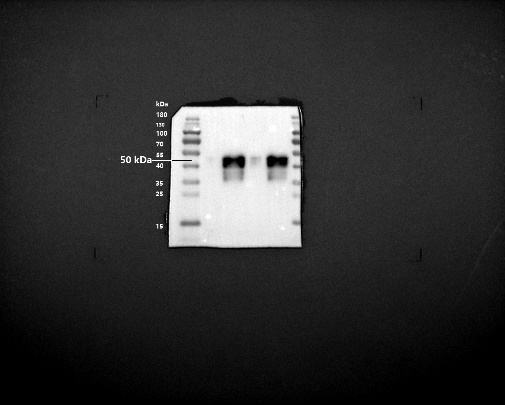


**TSG101 (Left two lane)**


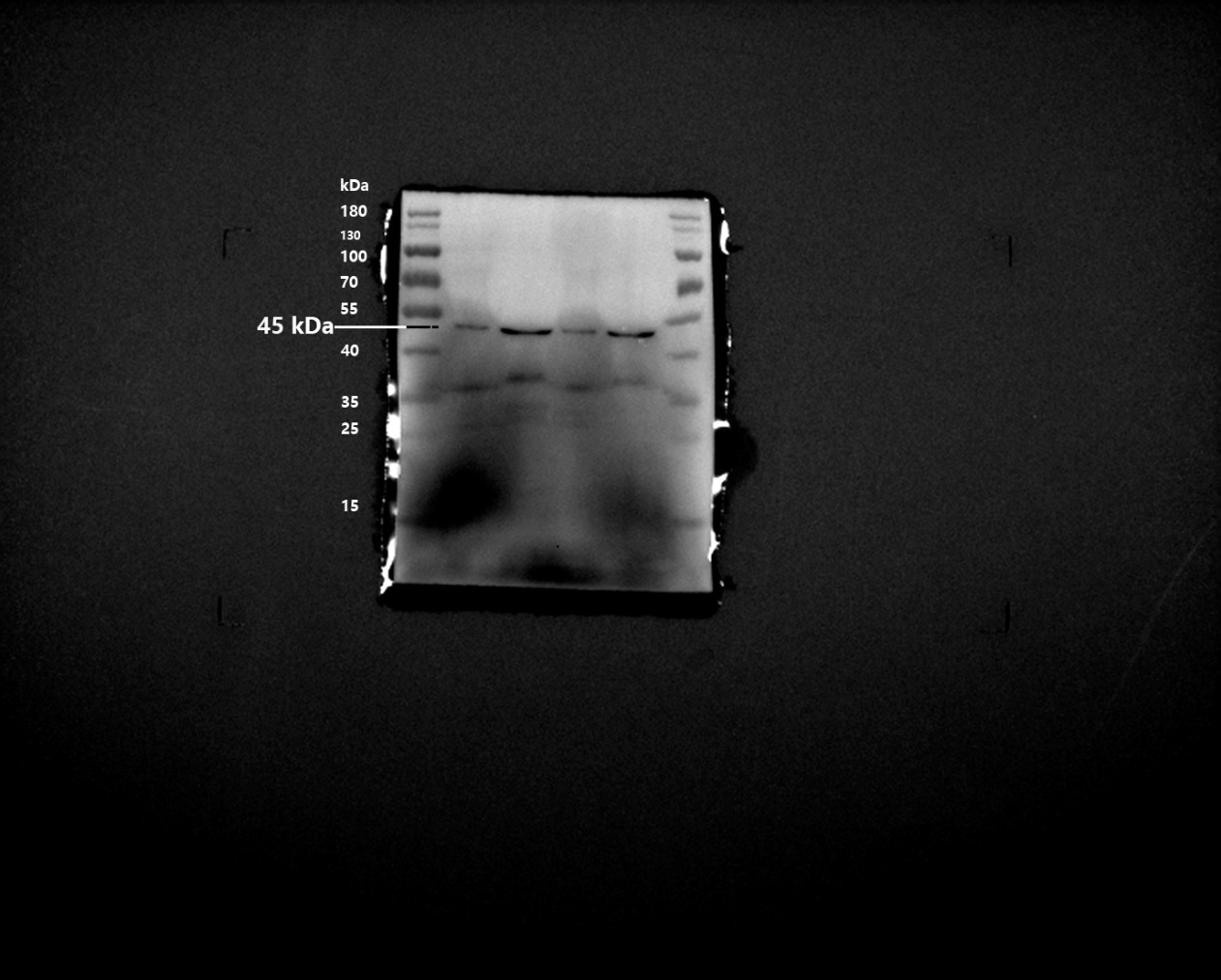


**GAPDH (Left two lane)**


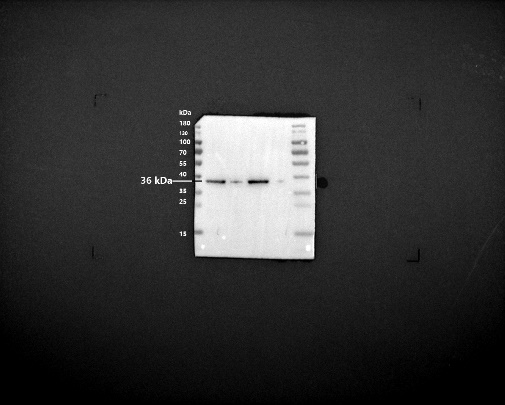


**Figure 4F.**

***Demonstrated pictures in Figure.***


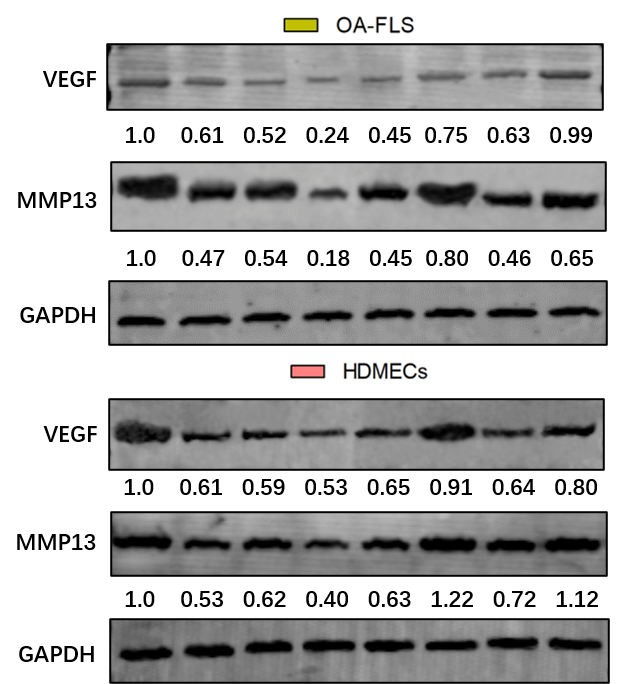


***Figure legends.***

Under incubation of IL-1β, VEGF/MMP13 protein expression in co-cultured OA-FLS/HDMECs were found to be significantly downregulated by OE-circPARD3B-SMSCs-Exos, and this downregulation could be significantly inhibited by miR-326-mimic-SMSCs-Exos.

***Original images***

**OA-FLS-VEGF**


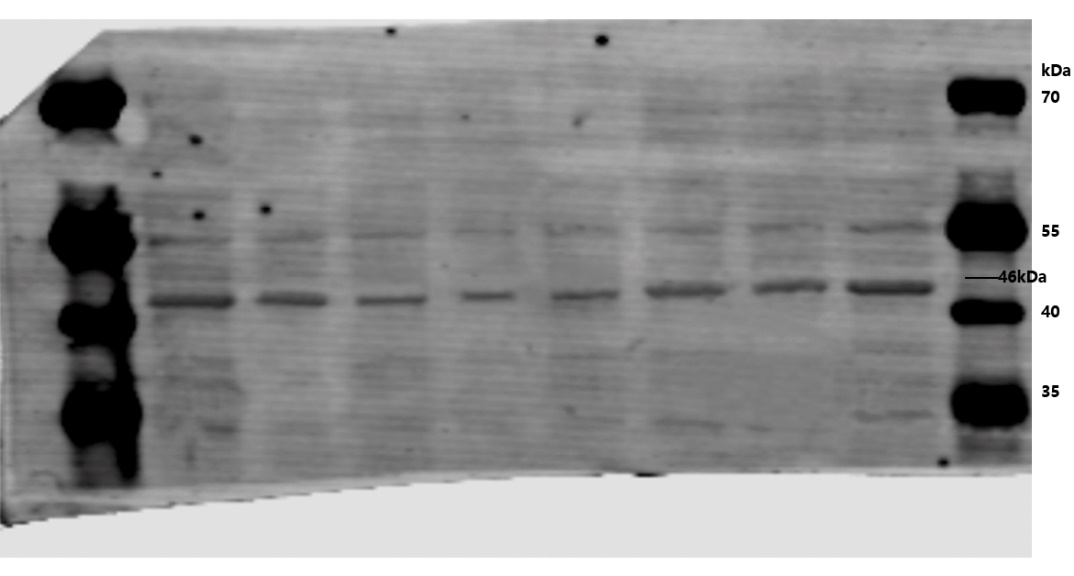


**OA-FLS-MMP13**


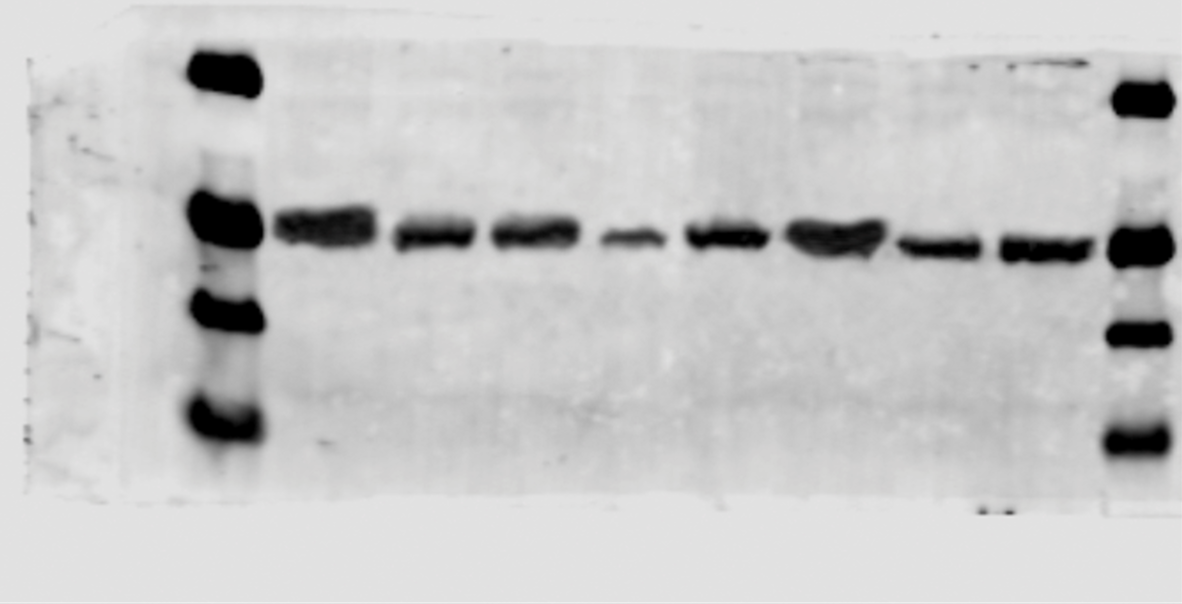


**OA-FLS-GAPDH**


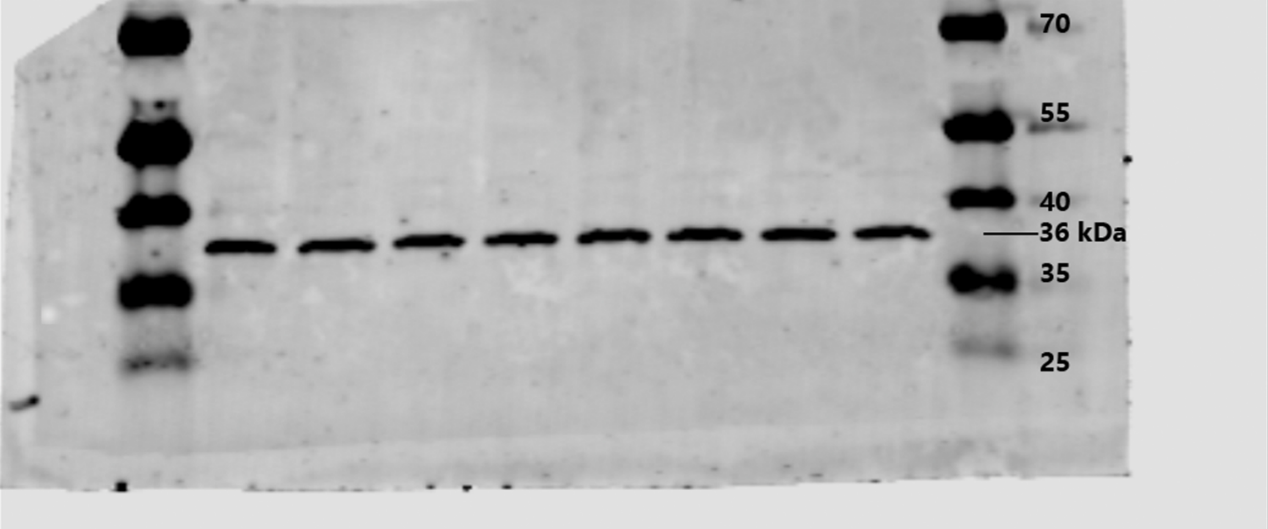


**HDMECs-VEGF**


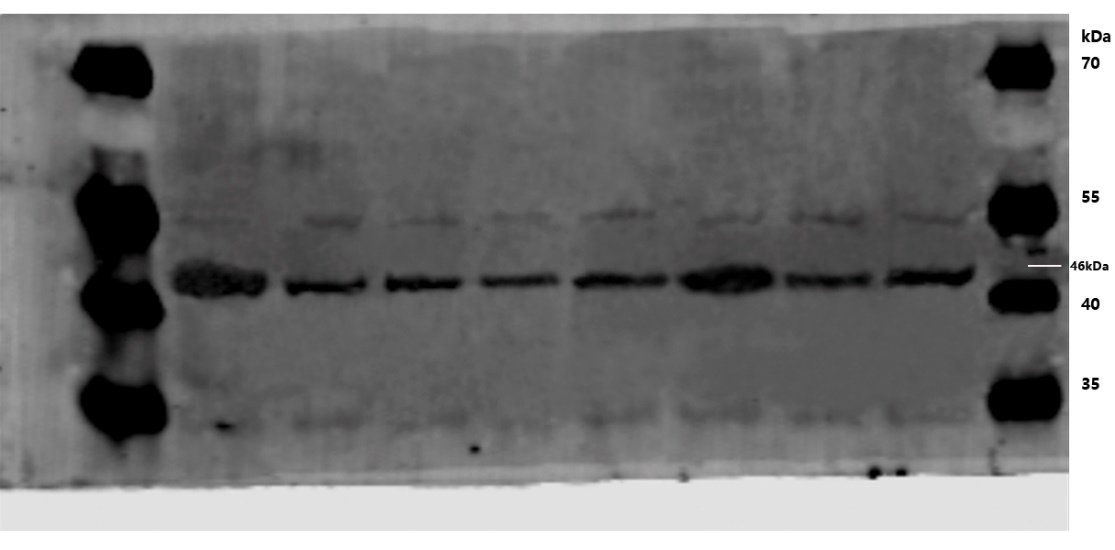


**HDMECs-MMP13**


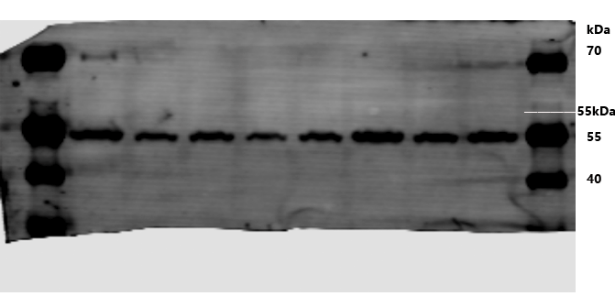


**HDMECs-GAPDH**


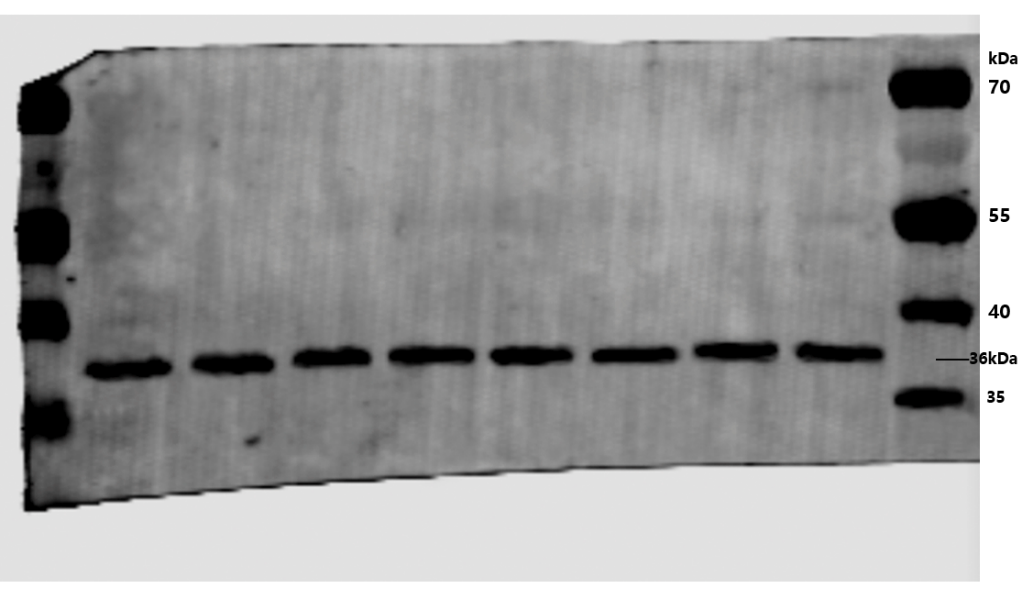


**Supplementary Figure 6B.**

***Demonstrated pictures in Figure.***

**
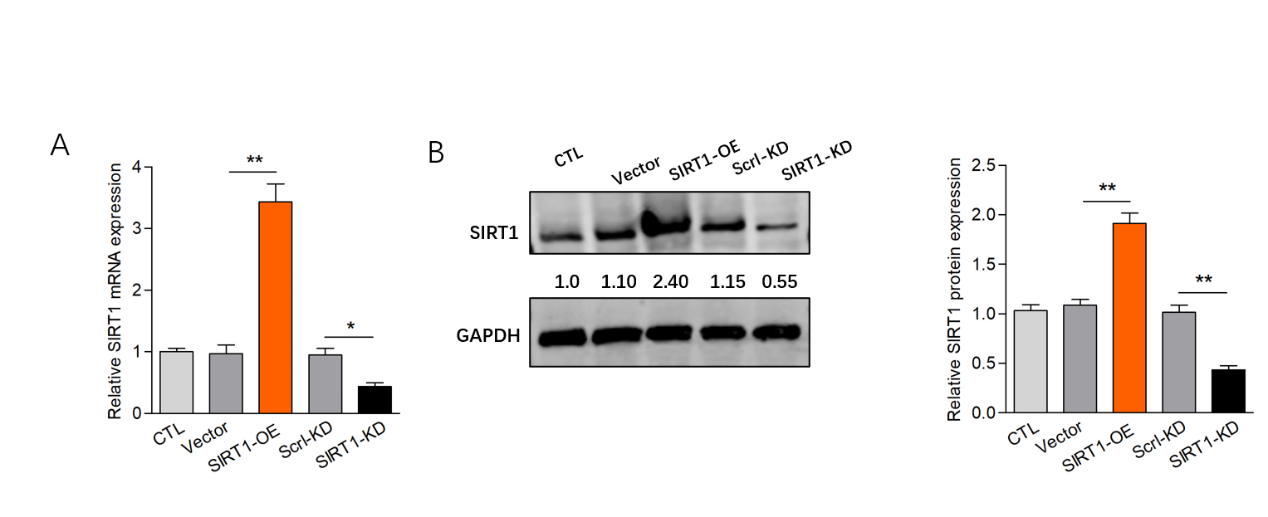
**

***Figure legends.***

SIRT1 protein expression were significantly upregulated by SIRT1 overexpression and downregulated by silencing.

***Original images***

**SIRT1**


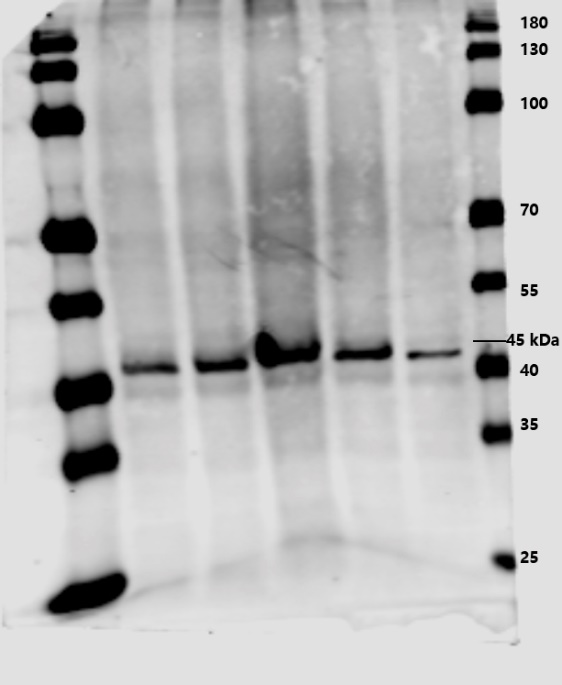


GAPDH


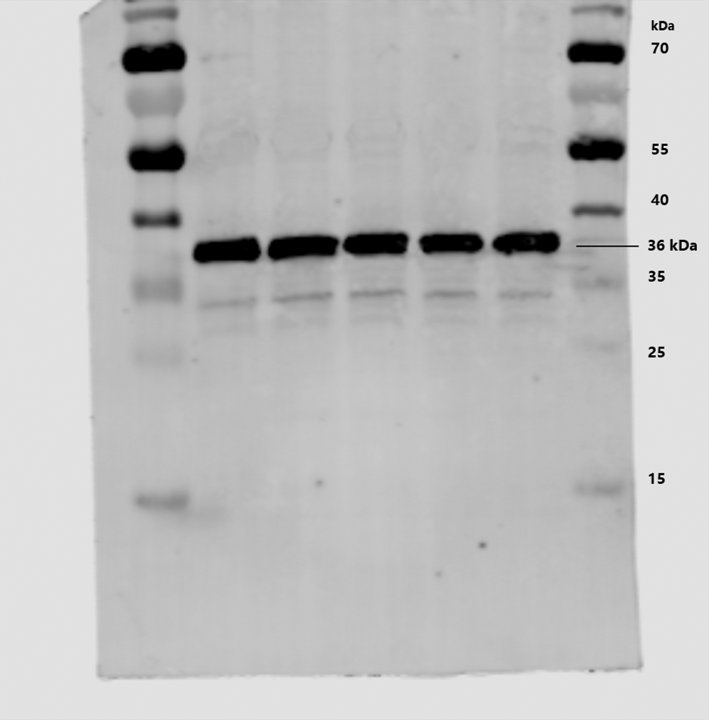

Supplement: Supplementary file 6 [file Table12.DOCX]
